# Tislelizumab plus chemotherapy for advanced or metastatic esophageal squamous-cell carcinoma in patients with PD-L1 Tumor Area Positivity score ≥5%: a *post hoc*, exploratory, long-term follow-up of RATIONALE-306^☆^

**DOI:** 10.1016/j.esmoop.2026.108362

**Published:** 2026-08-27

**Authors:** D. Tougeron, J. Xu, E. Raymond, R.A. Hubner, K. Kato, L. Wyrwicz, E. Van Cutsem, S.R. Park, P. Jimenez-Fonseca, T. Kojima, R. Ishihara, A. Avallone, R. Pazo-Cid, K. Wang, S. Yan, J. Shi, X. Kong, H.H. Yoon, F. Lordick

**Affiliations:** 1 Department of Hepato-Gastroenterology, CHU de Poitiers, Poitiers, France; 2 Department of Gastrointestinal Oncology, Fifth Medical Center, Chinese PLA General Hospital, Beijing, China; 3 Department of Medical Oncology & Hematology, Groupe Hospitalier Paris Saint-Joseph, Paris, France; 4 Department of Medical Oncology, The Christie NHS Foundation Trust, Manchester, UK; 5 Department of Head and Neck, Esophageal Medical Oncology, National Cancer Center Hospital, Tokyo, Japan; 6 Department of Oncology and Radiotherapy, Maria Sklodowska-Curie National Cancer Research Institute, Warsaw, Poland; 7 Digestive Oncology Division, University Hospitals Gasthuisberg, Leuven, Belgium; 8 Digestive Oncology, KU Leuven, Leuven, Belgium; 9 Department of Oncology, Asan Medical Center, University of Ulsan College of Medicine, Seoul, Republic of Korea; 10 Department of Medical Oncology, Hospital Universitario Central de Asturias, ISPA, Oviedo, Spain; 11 Department of Gastrointestinal Oncology, National Cancer Center Hospital East, Chiba, Japan; 12 Department of Gastrointestinal Oncology, Osaka International Cancer Institute, Osaka, Japan; 13 Experimental Clinical Abdominal Oncology Unit, Istituto Nazionale Tumori-IRCCS Fondazione G. Pascale, Naples, Italy; 14 Department of Medical Oncology, Hospital Universitario Miguel Servet, Zaragoza, Spain; 15 Global Statistics and Data Science, BeOne Medicines, Ltd, Ridgefield Park, USA; 16 Clinical Development, BeOne Medicines, Ltd, Beijing, China; 17 Clinical Biomarker, BeOne Medicines, Ltd, Beijing, China; 18 Clinical Development, BeOne Medicines, Ltd, Shanghai, China; 19 Department of Oncology, Mayo Clinic, Rochester, USA; 20 Department of Medicine (Oncology, Gastroenterology, Hepatology, and Pulmonology), University of Leipzig Medical Center, Comprehensive Cancer Center Central Germany, Leipzig, Germany

**Keywords:** esophageal squamous-cell carcinoma, programmed death-ligand 1 (PD-L1), tislelizumab

## Abstract

**Background:**

In the randomized, double-blind, phase III RATIONALE-306 trial, patients with unresectable, locally advanced, recurrent, or metastatic esophageal squamous-cell carcinoma (ESCC) treated with tislelizumab plus chemotherapy in the intent-to-treat population and in the programmed death-ligand 1 (PD-L1) Tumor Area Positivity (TAP) score ≥5% subgroup experienced clinically meaningful overall survival (OS) benefit compared with placebo plus chemotherapy at the interim analysis and minimum 3-year follow-up. We report *post hoc*, exploratory, longer-term outcomes at study closeout with minimum 45.2-month follow-up in patients with PD-L1 TAP score ≥5% per European Medicines Agency recommendation.

**Patients and methods:**

Patients were randomly assigned (1 : 1) to receive tislelizumab 200 mg or placebo every 3 weeks plus investigator-chosen chemotherapy. The primary endpoint was OS. Secondary endpoints included progression-free survival (PFS), objective response rate (ORR), duration of response (DoR), safety, and quality of life (QoL).

**Results:**

Of 649 randomly allocated patients, 55.2% had PD-L1 TAP score ≥5% (tislelizumab plus chemotherapy, *n* = 172; placebo plus chemotherapy, *n* = 186). At 45.2-month minimum follow-up, tislelizumab plus chemotherapy improved OS [median 19.1 versus 10.0 months; hazard ratio (HR) 0.61] and PFS (median 8.2 versus 5.5 months; HR 0.50) compared with placebo plus chemotherapy. ORR was 71.5% versus 41.4%; median DoR was 7.1 versus 5.4 months, respectively. QoL was similar overall between arms, with tislelizumab plus chemotherapy trending toward better global health and pain reduction. Any-grade treatment-related adverse events (TRAEs) occurred in 97.7% (tislelizumab plus chemotherapy) versus 98.4% (placebo plus chemotherapy) and grade ≥3 TRAEs in 70.2% versus 66.5%, respectively. The most common grade ≥3 TRAEs were decreased neutrophil count (35.1% versus 31.9%), anemia (13.5% versus 11.4%), and decreased white blood cell count (12.3% versus 17.8%). Treatment-emergent adverse events with ≥5% difference in incidence between arms at interim analysis decreased substantially after 12 months.

**Conclusion:**

First-line tislelizumab plus chemotherapy provided sustained and clinically meaningful efficacy benefits over placebo plus chemotherapy and was tolerable, with no new safety signals for patients with unresectable, locally advanced, or metastatic ESCC and PD-L1 TAP score ≥5%.

## Highlights

• The phase III RATIONALE-306 trial investigated first-line tislelizumab (anti-PD-1) plus chemotherapy in advanced/metastatic ESCC.
• We report *post hoc* efficacy/safety at study closeout (minimum 45.2-month follow-up) in patients with PD-L1 TAP score ≥5%.
• Tislelizumab plus chemotherapy improved OS, PFS, ORR, and DoR compared with placebo plus chemotherapy.
• Efficacy benefits were durable and maintained throughout the extended follow-up.
• The safety profile of tislelizumab plus chemotherapy remained manageable, with no new long-term signals.

## Introduction

Esophageal squamous-cell carcinoma (ESCC) is the predominant histologic subtype of esophageal cancer worldwide.^1^, ^2^, ^3^ Approximately 50% of patients are diagnosed with advanced disease, which carries a poor prognosis^4^ and highlights the need for effective treatments.

The efficacy and safety of programmed cell death protein 1 (PD-1) inhibitors in combination with chemotherapy, compared with chemotherapy alone, have been established in multiple trials.^5^, ^6^, ^7^, ^8^, ^9^, ^10^ In the RATIONALE-306 trial (NCT03783442; https://clinicaltrials.gov/study/NCT03783442, registered 21 December 2018), first-line tislelizumab (an anti-PD-1 antibody) plus chemotherapy demonstrated clinically meaningful improvement in overall survival (OS) compared with placebo plus chemotherapy after a minimum 3-year follow-up both in the intent-to-treat (ITT) population [hazard ratio (HR) 0.70] of patients with advanced or metastatic ESCC and in an exploratory analysis of patients with programmed death-ligand 1 (PD-L1) Tumor Area Positivity (TAP) score ≥5% (HR 0.63) [Yoon et al. (unpublished data)]. Based on positive outcomes, tislelizumab gained regulatory approval in multiple countries/regions as a first-line treatment in combination with chemotherapy for patients with advanced or metastatic ESCC with varying PD-L1 expression cut-offs.^11^^,^^12^ In 2024, the European Medicines Agency (EMA) approved first-line tislelizumab plus platinum-based chemotherapy specifically for patients with PD-L1 TAP score ≥5%.^13^

The TAP score is one method of determining PD-L1 expression level in oncology.^14^ Other approaches, such as combined positive score (CPS) and tumor cell (TC) score or tumor proportion score (TPS), are also used to quantify PD-L1 expression in tumor samples.^15^ An analysis combining patient samples from the RATIONALE-305 (gastric cancer/gastroesophageal junction cancer), RATIONALE-302 (ESCC), and RATIONALE-306 trials demonstrated high concordance between TAP score and CPS and similar correlations with clinical outcomes and safety across PD-L1 thresholds.^16^ Previously, another retrospective analysis of RATIONALE-306 found that tislelizumab plus chemotherapy provided clinically meaningful improvement in OS, progression-free survival (PFS), and objective response rate (ORR) in patient subgroups with PD-L1 TAP score ≥1% and PD-L1 CPS ≥1.^17^ Some therapeutic agents, such as pembrolizumab and nivolumab, have received approvals on the basis of PD-L1 positivity defined by specific methods, including TPS or CPS, depending on the indication.^18^^,^^19^

Given the need for clinical guidance, particularly regarding the use of tislelizumab in patients with PD-L1 TAP score ≥5%, and in accordance with the EMA recommendation, we conducted a *post hoc* analysis of long-term follow-up data (at study closeout) for this subgroup in RATIONALE-306.

## Patients and methods

### Study design and participants

RATIONALE-306 (NCT03783442) is a global, randomized, double-blind, placebo-controlled, phase III trial evaluating treatment in adults (≥18 years) with histologically confirmed, unresectable, locally advanced, recurrent, or metastatic ESCC. The study protocol, including eligibility criteria and endpoint assessments, was published by Xu et al.^10^ In brief, eligible patients had not received prior systemic therapy for advanced disease and were not candidates for definitive therapies, including surgery or chemoradiation, per local investigator evaluation/multidisciplinary team. Patients previously treated with neoadjuvant or adjuvant platinum-based chemotherapy were required to have a treatment-free interval of ≥6 months. Participants needed an Eastern Cooperative Oncology Group performance status of 0 or 1 and measurable disease per RECIST version 1.1. The study adhered to the International Council for Harmonisation Good Clinical Practice Guidelines, the Declaration of Helsinki, and applicable local regulations. All procedures complied with relevant laws and institutional guidelines and were approved by the appropriate institutional review boards or independent ethics committees (see Supplementary Table S1, available at https://doi.org/10.1016/j.esmoop.2026.108362, for full details). All patients provided informed consent, and safety was monitored by an independent data-monitoring committee. Patients and the public were not involved in the design, conduct, or reporting of this trial.

### Randomization and stratification

Patients were randomly assigned 1 : 1 to receive tislelizumab plus chemotherapy or placebo plus chemotherapy. Stratification factors included investigator-chosen chemotherapy (platinum plus fluoropyrimidine versus platinum plus paclitaxel), region [Asia (excluding Japan) versus Japan versus other regions], and history of previous definitive therapy (yes versus no).

### Procedures

Treatment consisted of intravenous tislelizumab 200 mg or matching placebo administered once every 3 weeks (day 1 of each 21-day cycle) combined with investigator-chosen doublet chemotherapy. Chemotherapy included a platinum (cisplatin 60-80 mg/m^2^ intravenously on day 1 or oxaliplatin 130 mg/m^2^ intravenously on day 1) plus a fluoropyrimidine [5-fluorouracil (5FU; 750-800 mg/m^2^ intravenously on days 1-5) or capecitabine (1000 mg/m^2^ orally twice daily on days 1-14)] or paclitaxel (175 mg/m^2^ intravenously on day 1).

### Study endpoints and assessments

The primary endpoint was OS, and secondary endpoints included PFS, ORR, duration of response (DoR), safety, and patient-reported outcomes (PROs). PFS, ORR, and DoR were assessed by the investigator per RECIST version 1.1. Although assessment of PD-L1 expression was not required for enrollment, PD-L1 expression was measured retrospectively by TAP score [VENTANA PD-L1 (SP263) assay (Roche Diagnostics, Indianapolis, IN)].^14^ TAP score was defined as the percentage of the tumor area (tumor and any desmoplastic stroma) covered by TCs with PD-L1 membrane staining at any intensity and tumor-associated immune cells with PD-L1 staining at any intensity, as estimated visually.^14^ Subsequently, pathologists in the central laboratory [Q Squared Solutions, Edinburgh, UK, and Q Squared Solutions (Beijing), Beijing, China] scored the same stained samples according to TC score (percentage of viable TCs with any membrane staining above background) for exploratory analysis.

### Statistical analysis

Full details of the statistical analysis for the RATIONALE-306 study have been described previously.^10^ Efficacy outcomes are reported for the ITT population, which included all randomly allocated patients. The safety population comprised all patients who received at least one dose of the study treatment. OS, PFS, and DoR were estimated by Kaplan–Meier methodology, with 95% confidence intervals (CIs) estimated by the Brookmeyer and Crowley method. In this descriptive, *post hoc* analysis, HRs were calculated using Cox regression models with treatment as the covariate and the same stratification factors stated previously. Landmark OS analyses at 9, 12, 18, 24, and 36 months were conducted. No adjustments for multiple comparisons were applied. OS, PFS, and DoR rates were estimated by Kaplan–Meier methodology with 95% CIs based on Greenwood’s formula. ORR, ORR differences, and odds ratios between arms were calculated using the Cochran–Mantel–Haenszel method, stratified by the same factors. Two-sided 95% CIs were computed using the Clopper–Pearson method. The two-sided Cochran–Mantel–Haenszel test was applied using the same stratification approach. Disease control rate was defined as the proportion of patients with best response of complete response, partial response, stable disease, or noncomplete response/nonprogressive disease per RECIST version 1.1. PROs were evaluated with the European Organisation for Research and Treatment of Cancer (EORTC) Quality of Life Questionnaire–Cancer 30 question module (QLQ-C30) and the EORTC Quality of Life Questionnaire–Oesophageal Cancer 18 question module (QLQ-OES18). Higher scores for Global Health Scale/Quality of Life (GHS/QoL) and physical functioning indicate a better outcome, whereas higher scores for symptoms indicate worse outcomes. Differences within and between the treatment arms from baseline at key clinically justifiable cycles 6 (week 15) and 8 (week 21) (short- and mid-term assessments) were calculated using a mixed model for repeated measures, with both EORTC QLQ-C30 and EORTC QLQ-OES18 scores as the response variable and treatment by study visit interaction, baseline mean score, and stratification factors as covariates. Within- and between-group comparisons were reported as differences in the least-squares mean (LSM) change from baseline, along with 95% CIs. A clinically meaningful change was defined as a five-point mean change from baseline.^20^, ^21^, ^22^ The between-group *P* values were two-sided and nominal, without multiplicity adjustment. Efficacy and safety data were analyzed descriptively. All calculations and analyses were carried out using SAS version 9.4 (SAS Institute, Cary, NC).

## Results

### Patients

**Figure 1:**
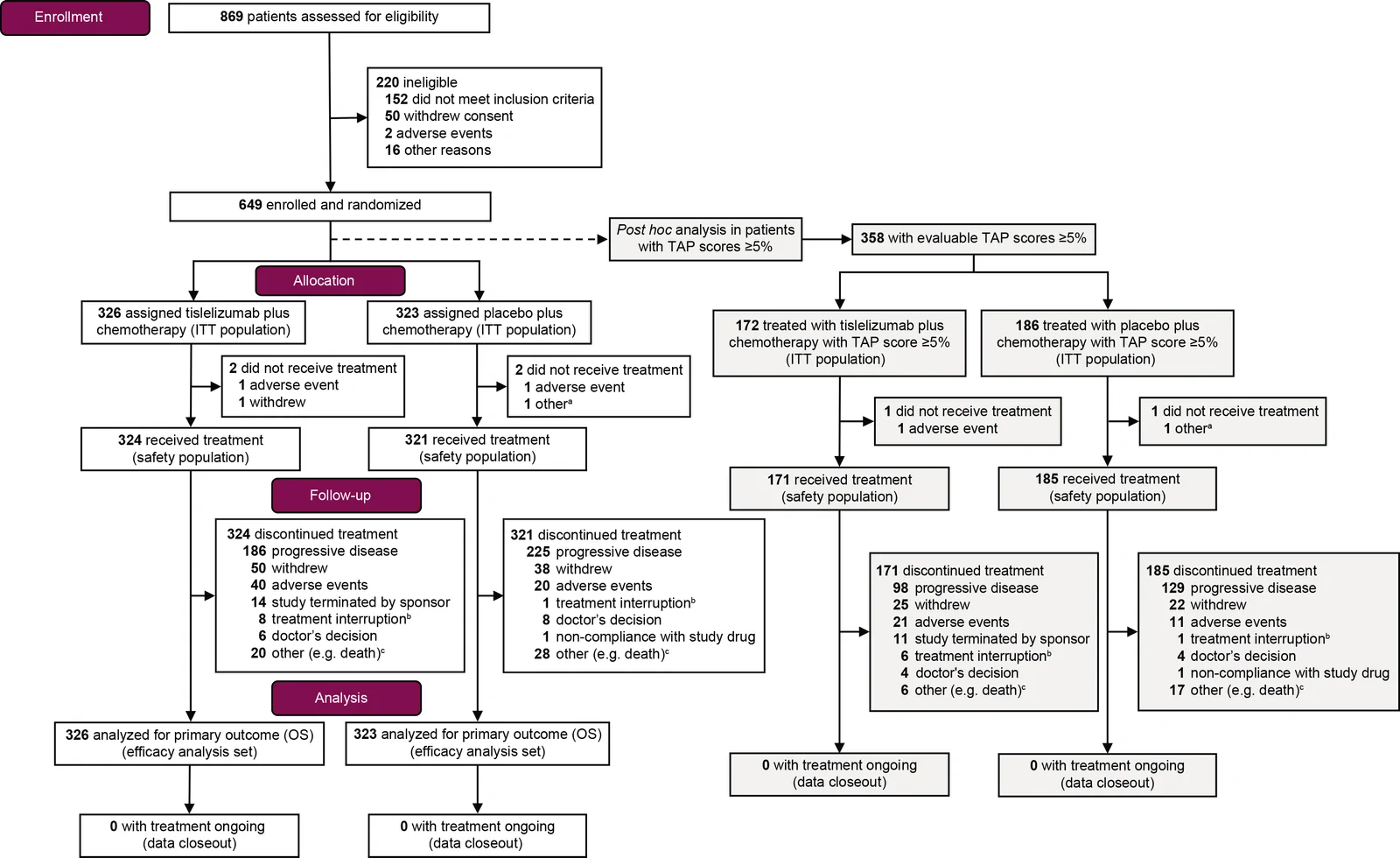
**Patient disposition at study closeout.** Study closeout (22 August 2024 cut-off). ^a^One patient not treated because of an interactive web response system technical issue. ^b^Patients with confirmed complete response, partial response, or stable disease after 2 years of study treatment could stop the treatment. ^c^Death was the main (but not only) reason for treatment discontinuation. ITT, intent-to-treat; OS, overall survival; TAP, Tumor Area Positivity.

Efficacy endpoints, including OS and PFS, were based on the final study closeout (22 August 2024 cut-off; minimum follow-up of 45.2 months). A total of 649 patients were randomly assigned to receive tislelizumab plus chemotherapy (*n* = 326) or placebo plus chemotherapy (*n* = 323) and comprised the ITT population. Overall, 358 patients (55%) had a PD-L1 TAP score ≥5%, 172 patients in the tislelizumab plus chemotherapy arm and 186 in the placebo plus chemotherapy arm. In the subgroup of patients with PD-L1 TAP score ≥5%, 78.2% were enrolled in Asia. One patient from each treatment arm did not receive treatment (Figure 1). Baseline demographics and clinical characteristics for patients with PD-L1 TAP score ≥5% were generally well balanced between treatment arms (Supplementary Table S2, available at https://doi.org/10.1016/j.esmoop.2026.108362). The baseline characteristics of patients with PD-L1 TAP score ≥5% were also consistent with those of the ITT population.^10^ Treatment exposure was similar between arms (Supplementary Tables S3 and S4, available at https://doi.org/10.1016/j.esmoop.2026.108362). Exposure to chemotherapy was in general similar between treatment arms for chemotherapy doublets A (platinum plus 5FU), B (platinum plus capecitabine), and C (platinum plus paclitaxel) (Supplementary Tables S5-S7, available at https://doi.org/10.1016/j.esmoop.2026.108362).

### Efficacy

**Figure 2:**
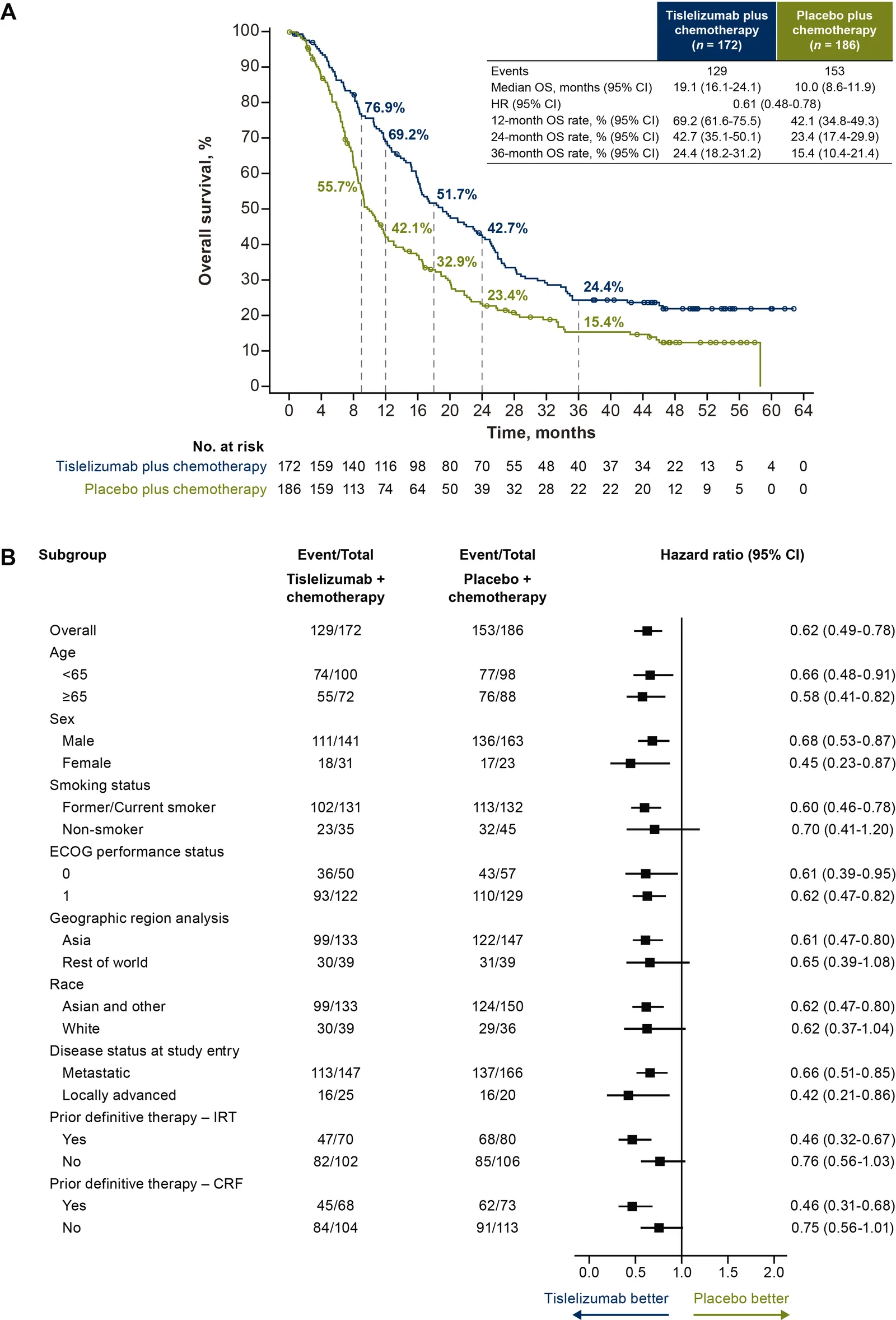
**(A) Kaplan–Meier plot of OS in patients with PD-L1 TAP score ≥5%; (B) forest plot of the prespecified subgroup analysis of OS.** Data in this figure are from *post hoc* analyses. The 9-month and 18-month OS rates for tislelizumab plus chemotherapy versus placebo plus chemotherapy were 76.9% (95% CI 69.8-82.6) versus 55.7% (95% CI 48.1-62.6) and 51.7% (95% CI 43.9-59.0) versus 32.9% (95% CI 26.1-39.9), respectively. The race subcategory Other includes American Indian or Alaska native, not reported, and unknown. PD-L1 status was determined using VENTANA PD-L1 (SP263) Assay. Unknown refers to patients without sample collection or not assessable at baseline. CI, confidence interval; CRF, case report form; ECOG, Eastern Cooperative Oncology Group; HR, hazard ratio; IRT, interactive response technology; OS, overall survival; PD-L1, programmed death-ligand 1; TAP, Tumor Area Positivity.

At a minimum follow-up of 45.2 months, tislelizumab plus chemotherapy improved OS in patients with PD-L1 TAP score ≥5%, with a median OS of 19.1 months (95% CI 16.1-24.1) compared with 10.0 months (95% CI 8.6-11.9) with placebo plus chemotherapy (HR 0.61, 95% CI 0.48-0.78; Figure 2A). OS rates at 9, 12, 18, 24, and 36 months were higher in the tislelizumab plus chemotherapy arm (76.9%, 69.2%, 51.7%, 42.7%, and 24.4%, respectively) than in the placebo plus chemotherapy arm (55.7%, 42.1%, 32.9%, 23.4%, and 15.4%, respectively). OS benefit with tislelizumab plus chemotherapy was consistent across all prespecified subgroups (Figure 2B).

**Figure 3:**
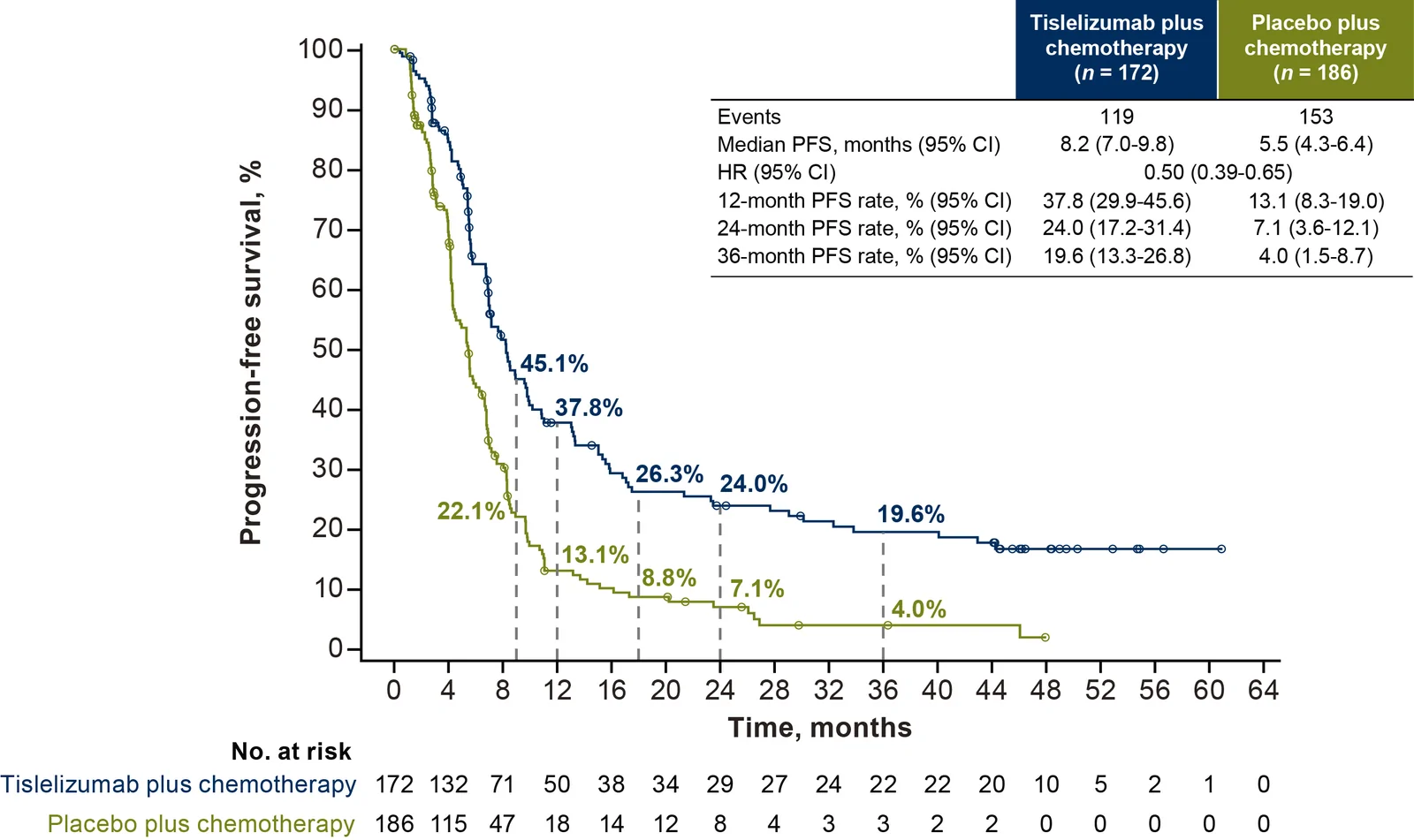
**Kaplan–Meier plot of PFS in patients with PD-L1 TAP score ≥5%.** Data in this figure are from *post hoc* analyses. The 9-month and 18--month PFS rates for tislelizumab plus chemotherapy versus placebo plus chemotherapy were 45.1% (95% CI 36.9-52.9) versus 22.1% (95% CI 16.0-28.9) and 26.3% (95% CI 19.3-33.8) versus 8.8% (95% CI 4.9-14.0), respectively. CI, confidence interval; HR, hazard ratio; PD-L1, programmed death-ligand 1; PFS, progression-free survival; TAP, Tumor Area Positivity.

At a minimum follow-up of 45.2 months, treatment with tislelizumab plus chemotherapy improved PFS compared with placebo plus chemotherapy in patients with PD-L1 TAP score ≥5%, with a median of 8.2 months (95% CI 7.0-9.8) versus 5.5 months (95% CI 4.3-6.4), respectively (HR 0.50, 95% CI 0.39-0.65; Figure 3). PFS rates at 9, 12, 18, 24, and 36 months were consistently higher in the tislelizumab plus chemotherapy arm (45.1%, 37.8%, 26.3%, 24.0%, and 19.6%, respectively) than in the placebo plus chemotherapy arm (22.1%, 13.1%, 8.8%, 7.1%, and 4.0%, respectively).

**Figure 4:**
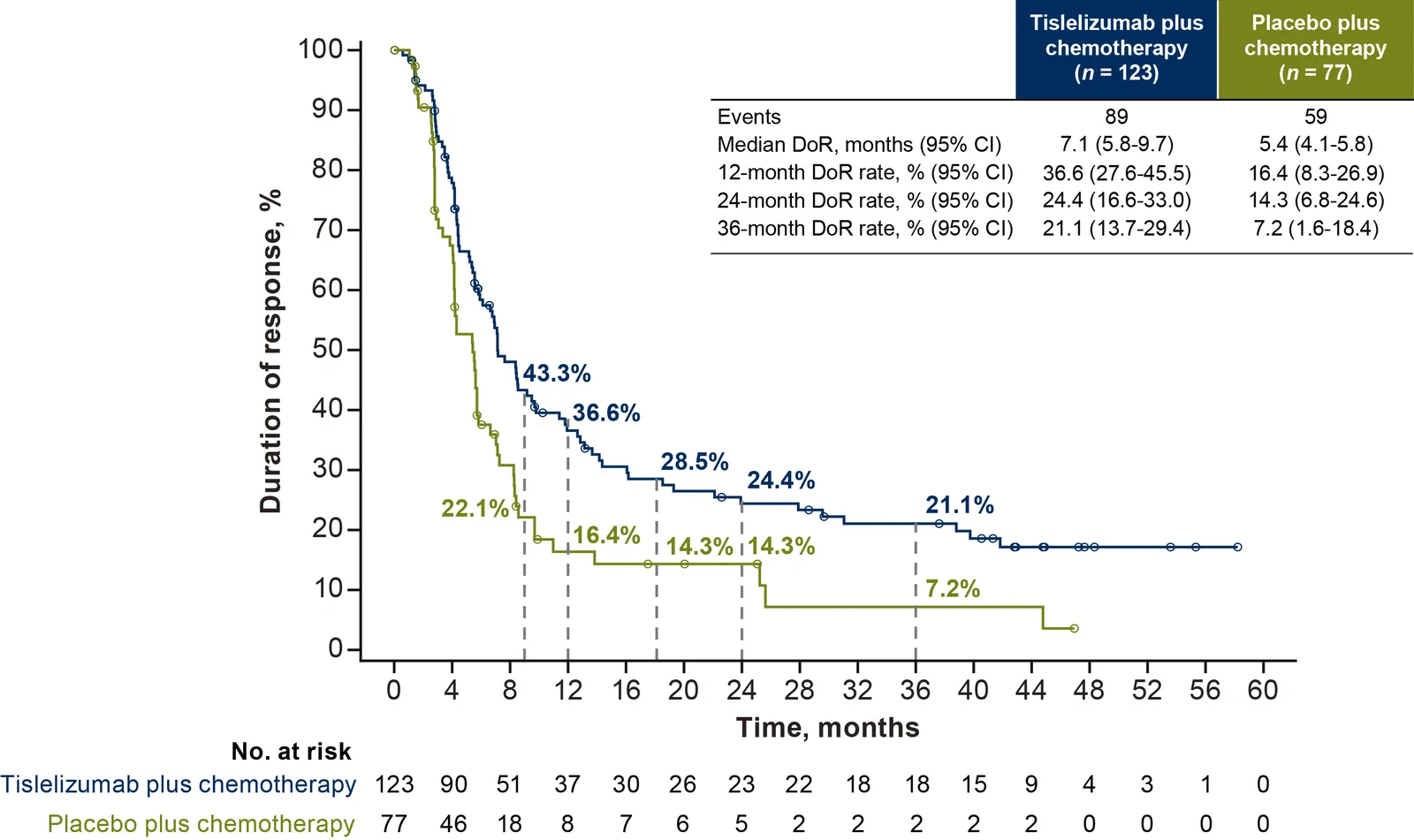
**Kaplan–Meier plot of DoR by investigator assessment in patients with PD-L1 TAP score ≥5%.** Data in this figure are from *post hoc* analyses. The 9-month and 18-month DoR rates for tislelizumab plus chemotherapy versus placebo plus chemotherapy were 43.3% (95% CI 34.0-52.3) versus 22.1% (95% CI 12.8-33.0) and 28.5% (95% CI 20.3-37.3) versus 14.3% (95% CI 6.8-24.6), respectively. CI, confidence interval; DoR, duration of response; PD-L1, programmed death-ligand 1; TAP, Tumor Area Positivity.

At a minimum follow-up of 45.2 months, the ORR was higher in patients with PD-L1 TAP score ≥5% treated with tislelizumab plus chemotherapy compared with placebo plus chemotherapy [71.5% (95% CI 64.1% to 78.1%) versus 41.4% (95% CI 34.2% to 48.8%)]. Response data were consistent at the minimum 3-year follow-up and minimum 45.2-month follow-up (Supplementary Tables S8 and S9, available at https://doi.org/10.1016/j.esmoop.2026.108362). Complete response was observed in 7.0% and partial response in 64.5% of patients in the tislelizumab plus chemotherapy arm, compared with 1.6% and 39.8%, respectively, in the placebo plus chemotherapy arm. Median DoR was 7.1 months (95% CI 5.8-9.7) with tislelizumab plus chemotherapy versus 5.4 months (95% CI 4.1-5.8) with placebo plus chemotherapy (Figure 4). Landmark DoR rates at 9, 12, 18, 24, and 36 months were consistently higher with tislelizumab plus chemotherapy (43.3%, 36.6%, 28.5%, 24.4%, and 21.1%, respectively) than with placebo plus chemotherapy (22.1%, 16.4%, 14.3%, 14.3%, and 7.2%, respectively).

Exploratory analysis of TC score subgroups within the population of patients with tumors with PD-L1 TAP score ≥5% is available in Supplementary Table S10, Supplementary Results, and Supplementary Discussion (available at https://doi.org/10.1016/j.esmoop.2026.108362).

### Subsequent anticancer therapy

Among patients with PD-L1 TAP score ≥5%, 55.2% in the tislelizumab plus chemotherapy arm and 61.3% in the placebo plus chemotherapy arm received subsequent systemic therapy. The most frequent were radiotherapy (21.5% versus 25.3%) and immunotherapy (15.7% versus 23.7%), respectively (Supplementary Table S11, available at https://doi.org/10.1016/j.esmoop.2026.108362). The mean [standard deviation (SD)] duration of subsequent systemic therapy was 7.7 (8.6) months and 7.5 (9.9) months for tislelizumab plus chemotherapy and placebo plus chemotherapy, respectively mean (SD) duration of immunotherapy was 3.6 (4.0) and 6.0 (8.5) months, respectively.

### PROs

At cycle 6 (week 15), the LSM change from baseline was maintained for the QLQ-C30 GHS/QoL scale in the tislelizumab plus chemotherapy arm, whereas deterioration occurred in the placebo plus chemotherapy arm [LSM change 1.5 (95% CI −1.4 to 4.4) versus −3.1 (95% CI −6.1 to −0.0)], with a significant between-group difference in favor of tislelizumab plus chemotherapy observed (LSM difference 4.5, 95% CI 0.6-8.5, nominal *P* = 0.0258). Both treatment arms experienced worsening in physical functioning, but the decline was less in the tislelizumab plus chemotherapy arm than in the placebo plus chemotherapy arm, in which clinically meaningful worsening was observed [LSM change −4.0 (95% CI −6.4 to −1.6) versus −8.3 (95% CI −10.8 to −5.8)]. The LSM treatment difference for the physical function scale was significant in favor of the tislelizumab plus chemotherapy arm (LSM difference 4.3, 95% CI 1.0-7.7, nominal *P* = 0.0110).

For the QLQ-OES18 pain scale at cycle 6, clinically meaningful improvement was observed in the tislelizumab plus chemotherapy arm compared with the placebo plus chemotherapy arm [LSM change −5.9 (95% CI −7.9 to −4.0) versus −2.7 (95% CI −4.7 to −0.6)], with a significant between-group difference in favor of tislelizumab plus chemotherapy (LSM difference −3.2, 95% CI −5.8 to −0.6, nominal *P* = 0.0156). Fatigue worsened similarly in both arms (Supplementary Figure S1, available at https://doi.org/10.1016/j.esmoop.2026.108362).

For the QLQ-C30 by cycle 8 (week 21), greater improvement from baseline in GHS/QoL was observed in the tislelizumab plus chemotherapy arm, with maintenance observed in the placebo plus chemotherapy arm [LSM change 3.2 (95% CI 0.4-6.0) versus −0.3 (95% CI −3.4 to 2.8)]; differences were no longer significant between the arms. Both arms worsened in physical functioning by cycle 8, with less clinically meaningful worsening observed in the tislelizumab plus chemotherapy compared with the placebo plus chemotherapy arm [LSM change −4.1 (95% CI −6.4 to −1.8) versus −7.0 (95% CI −9.5 to −4.5)]; no significant differences were observed between the arms (Supplementary Figure S2, available at https://doi.org/10.1016/j.esmoop.2026.108362).

For the QLQ-OES18 by cycle 8, the index score and reflux scores were maintained in both arms; no differences were observed between arms. For dysphagia, the placebo plus chemotherapy arm (LSM change −11.9, 95% CI −18.7 to −5.1) experienced a clinically meaningful improvement compared with the tislelizumab plus chemotherapy arm (LSM change −2.3, 95% CI −8.3 to 3.6), with a significant between-group difference observed (LSM difference 9.6, 95% CI 0.9-18.3, *P* = 0.0305). For pain by cycle 8, the tislelizumab plus chemotherapy arm continued to experience a clinically meaningful reduction in mean pain symptoms compared with the placebo plus chemotherapy arm [LSM change −5.2 (95% CI −7.3 to −3.1) versus −2.8 (95% CI −5.1 to −0.5)]; no additional significant differences were observed between the arms.

### Safety and tolerability

Among patients with PD-L1 TAP score ≥5%, treatment-related adverse events (TRAEs) of any grade were reported by 97.7% of those receiving tislelizumab plus chemotherapy and 98.4% of those treated with placebo plus chemotherapy. Grade ≥3 TRAEs occurred in 70.2% and 66.5% of patients, respectively (Table 1), and serious TRAEs were reported in 29.2% and 20.5%, respectively. The most frequently reported grade ≥3 TRAEs were decreased neutrophil count (35.1% versus 31.9%), anemia (13.5% versus 11.4%), and decreased white blood cell count (12.3% versus 17.8%).

**Table 1:** TRAEs occurring in ≥10% of patients with PD-L1 TAP score ≥5% in either treatment arm who received at least one dose of study treatment (safety population)

TRAE	Tislelizumab plus chemotherapy (*n* = 171)		Placebo plus chemotherapy (*n* = 185)	
	All grades	Grade ≥3	All grades	Grade ≥3
Patients with ≥1 TRAE	167 (97.7)	120 (70.2)	182 (98.4)	123 (66.5)
Anemia	98 (57.3)	23 (13.5)	87 (47.0)	21 (11.4)
Neutrophil count decreased	90 (52.6)	60 (35.1)	89 (48.1)	59 (31.9)
White blood cell count decreased	87 (50.9)	21 (12.3)	94 (50.8)	33 (17.8)
Decreased appetite	63 (36.8)	4 (2.3)	67 (36.2)	5 (2.7)
Nausea	56 (32.7)	4 (2.3)	86 (46.5)	3 (1.6)
Alopecia	40 (23.4)	0	34 (18.4)	0
Peripheral sensory neuropathy	38 (22.2)	7 (4.1)	37 (20.0)	4 (2.2)
Platelet count decreased	37 (21.6)	6 (3.5)	32 (17.3)	2 (1.1)
Diarrhea	33 (19.3)	5 (2.9)	34 (18.4)	3 (1.6)
Stomatitis	32 (18.7)	5 (2.9)	23 (12.4)	4 (2.2)
Vomiting	31 (18.1)	3 (1.8)	47 (25.4)	4 (2.2)
Weight decreased	30 (17.5)	0	26 (14.1)	0
Constipation	29 (17.0)	0	22 (11.9)	0
Fatigue	26 (15.2)	6 (3.5)	35 (18.9)	4 (2.2)
Aspartate aminotransferase increased	25 (14.6)	5 (2.9)	11 (5.9)	0
Malaise	24 (14.0)	1 (0.6)	33 (17.8)	1 (0.5)
Neutropenia	24 (14.0)	7 (4.1)	30 (16.2)	18 (9.7)
Alanine aminotransferase increased	23 (13.5)	4 (2.3)	16 (8.6)	1 (0.5)
Blood creatinine increased	21 (12.3)	0	15 (8.1)	0
Hypoesthesia	21 (12.3)	0	27 (14.6)	1 (0.5)
Hyponatremia	21 (12.3)	9 (5.3)	26 (14.1)	9 (4.9)
Hypothyroidism	21 (12.3)	0	7 (3.8)	0
Rash	21 (12.3)	4 (2.3)	14 (7.6)	0
Asthenia	18 (10.5)	2 (1.2)	25 (13.5)	1 (0.5)
Hypokalemia	18 (10.5)	10 (5.8)	19 (10.3)	8 (4.3)

Data are presented as *n* (%).

Presented TRAE data include TRAEs that occurred in ≥10% of patients in either treatment arm who received at least one dose of study treatment. Patients with multiple events for a given preferred term and system organ class are counted only once at the worst severity for the preferred term and system organ class, respectively. Adverse event terms were coded using the Medical Dictionary for Regulatory Activities version 24.0. TRAEs include TEAEs that were considered by the investigator to be related to study treatments or TEAEs with a missing causality.

PD-L1, programmed death-ligand 1; TAP, Tumor Area Positivity; TEAE, treatment-emergent adverse event; TRAE, treatment-related adverse event.

TRAEs leading to death were reported in 2.9% of patients in the tislelizumab plus chemotherapy arm and 1.6% of patients in the placebo plus chemotherapy arm (Supplementary Table S12, available at https://doi.org/10.1016/j.esmoop.2026.108362). Treatment discontinuation owing to TRAEs was more frequent with tislelizumab (32.2% versus 18.4%), most frequently because of peripheral sensory neuropathy (9.4% versus 3.2%) and hypoesthesia (2.9% versus 2.2%) (Supplementary Table S13, available at https://doi.org/10.1016/j.esmoop.2026.108362).

No immune-mediated adverse events led to death in either arm (Supplementary Table S14, available at https://doi.org/10.1016/j.esmoop.2026.108362). The most frequently reported any-grade immune-mediated adverse events with tislelizumab plus chemotherapy versus placebo plus chemotherapy were hypothyroidism (13.5% versus 3.8%), rash (12.3% versus 7.6%), pneumonitis (7.0% versus 3.2%), and hyperthyroidism (4.1% versus 1.1%).

Selected treatment-emergent adverse events (TEAEs) with ≥5% difference between arms at the interim analysis (median follow-up of 16.3 months^10^) were included in this analysis to assess trends in their incidence over longer time periods (Supplementary Table S15, available at https://doi.org/10.1016/j.esmoop.2026.108362). At 12 months, the incidence rates for tislelizumab plus chemotherapy versus placebo plus chemotherapy were 40.9% versus 40.0% for decreased appetite, 21.6% versus 29.2% for vomiting, 11.7% versus 5.9% for pruritus, and 12.9% versus 3.8% for hypothyroidism. The incidence of all selected TEAEs decreased substantially after 12 months in both arms. By 12-24 months in the tislelizumab plus chemotherapy arm, rates of decreased appetite (13.5%), vomiting (3.8%), pruritus (5.8%), and hypothyroidism (1.9%) had dropped substantially compared with rates at ≤12 months (40.9%, 21.6%, 11.7%, and 12.9%, respectively).

## Discussion

This exploratory *post hoc* analysis describes long-term clinical efficacy and safety findings from RATIONALE-306 in patients with unresectable, locally advanced, recurrent, or metastatic ESCC and PD-L1 TAP score ≥5%. At a minimum follow-up of 45.2 months, patients who received tislelizumab plus chemotherapy experienced clinically meaningful and sustained improvements in OS, PFS, and ORR compared with those who received placebo plus chemotherapy. These findings, which are consistent with both the interim analysis (median follow-up of 16.3 months)^10^ and the 3-year follow-up results (minimum follow-up of 36.0 months),^23^ further support the long-term efficacy of tislelizumab plus chemotherapy as a first-line treatment option in this population.

This exploratory, *post hoc* analysis of the PD-L1 TAP score ≥5% subgroup, although not prespecified in the RATIONALE-306 trial design, provides mature efficacy and safety data at the 5% regulatory approval cut-off, enabling clinicians to assess the durability and sustainability of treatment benefit over time and supporting more informed treatment decisions in clinical practice. The pronounced and durable efficacy observed in the PD-L1 TAP score ≥5% subgroup provides important clinical context for understanding differential treatment benefit in advanced ESCC. Rather than redefining the biomarker landscape, these findings reinforce the relevance of PD-L1 TAP score as an enrichment strategy to identify patients who are more likely to derive enhanced benefit from tislelizumab-based chemo-immunotherapy. The findings are consistent with the broader shift toward biomarker-enriched first-line immunotherapy strategies in ESCC, as illustrated by the EMA approval of first-line tislelizumab plus platinum-based chemotherapy for advanced ESCC with PD-L1 TAP score ≥5%.

The safety profile of tislelizumab plus chemotherapy remained consistent with previous ITT population reports, with no new safety signals identified.^10^^,^^23^ Of note, selected TEAEs that showed ≥5% incidence differences between arms at the interim analysis^10^ (appetite loss, vomiting, pruritus, and hypothyroidism) all decreased substantially after 12 months in both arms, suggesting limited risk of cumulative toxicity over time.

Studies on PROs following treatment with anti-PD-1 monoclonal antibodies in ESCC are limited. Our study found improvement at cycle 6 with tislelizumab plus chemotherapy compared with placebo plus chemotherapy for EORTC QLQ-C30 GHS/QoL and physical functioning as well as for QLQ-OES18 pain. At cycle 8, the tislelizumab plus chemotherapy arm had greater improvement for GHS/QoL, reduced worsening for physical functioning, and reduced pain, although between-arm differences were not significant. These findings appear directionally more favorable than those observed in the KEYNOTE-590 study, in which all PRO endpoints, including QLQ-OES18, remained stable over 18 weeks, with no difference between treatment arms in patients with ESCC.^24^ Collectively, these findings indicate that the long-term clinical benefit of tislelizumab plus chemotherapy is achieved without compromising health-related QoL.

The results of this *post hoc* analysis of patients with PD-L1 TAP score ≥5% should be considered in the context of a few limitations. The analysis is descriptive in nature and was not prespecified in the trial protocol and is therefore subject to biases associated with *post hoc* analyses. Notably, the relatively large dataset (*n* = 358) reduces the risk typically associated with smaller exploratory analyses. However, the small sample sizes at later time points make accurate interpretation of efficacy results difficult. The majority of patients (78.2%) in the PD-L1 TAP score ≥5% subgroup were enrolled in Asia, which may limit generalizability to European populations, although a recent meta-analysis of 12 randomized controlled trials in advanced/metastatic ESCC demonstrated no statistically significant difference in OS between Asian (HR 0.70, 95% CI 0.66-0.75) and non-Asian (HR 0.73, 95% CI 0.63-0.85) subgroups receiving first- or second-line anti-PD-(L)1 therapy.^25^

In conclusion, this extended long-term follow-up *post hoc* analysis of patients with unresectable, locally advanced, or metastatic ESCC and PD-L1 TAP score ≥5% in the RATIONALE-306 trial demonstrated sustained improvements in efficacy with first-line tislelizumab plus chemotherapy over placebo plus chemotherapy. Tislelizumab plus chemotherapy was well tolerated, and the safety profile was manageable and consistent with that in the overall population. Together, these findings reinforce the clinical value of tislelizumab-based chemo-immunotherapy in advanced ESCC and support the continued refinement of biomarker-enriched strategies to optimize patient selection.
