# Supplementary material for: Tislelizumab plus chemotherapy for advanced or metastatic esophageal squamous-cell carcinoma in patients with PD-L1 Tumor Area Positivity score ≥5%: a post hoc, exploratory, long-term follow-up of RATIONALE-306☆
Source: ESMO Open. 2026 Aug 27;11(9):108362. doi: 10.1016/j.esmoop.2026.108362 (PMC13546845; doi:10.1016/j.esmoop.2026.108362)
Supplement: Supplementary file 1 [file mmc1.docx]

# Supplementary Material for Tougeron D, et al. Tislelizumab plus chemotherapy for advanced or metastatic esophageal squamous cell carcinoma in patients with PD-L1 Tumor Area Positivity score ≥5%: a post-hoc, exploratory, long-term follow-up of RATIONALE-306

## Supplementary Results

**PD-L1 TAP score ≥5% by TC score subgroup analysis**

Within the subgroup of patients with PD-L1 TAP score ≥5%, 133 of 172 (77%) patients in the tislelizumab plus chemotherapy arm and 125 of 186 (67%) patients in the placebo plus chemotherapy arm also had PD-L1 TC score ≥1%.

In patients with both PD-L1 TAP score ≥5% and PD-L1 TC score ≥1%, median OS was 17.2 months (95% CI 15.3-22.9) among those treated with tislelizumab plus chemotherapy and 10.6 months (95% CI 8.6-14.2) among those who received placebo plus chemotherapy (HR 0.68, 95% CI 0.51-0.90). Median PFS was 8.2 months (95% CI 7.0-10.0) with tislelizumab plus chemotherapy versus 5.5 months (95% CI 4.3-6.5) with placebo plus chemotherapy (HR 0.52, 95% CI 0.39-0.70). ORR was 70.7% (95% CI 62.2%-78.2%) with tislelizumab plus chemotherapy versus 36.8% (95% CI 28.4%-45.9%) with placebo plus chemotherapy (Supplementary Table S10).

Among patients with PD-L1 TAP score ≥5% and TC score <1% (*n* = 39 tislelizumab plus chemotherapy; *n* = 59 placebo plus chemotherapy), median OS was 25.1 months (95% CI 15.2-33.8) among those treated with tislelizumab plus chemotherapy versus 9.8 months (95% CI 8.0-12.3) in those who received placebo plus chemotherapy (HR 0.47, 95% CI 0.28-0.79). Median PFS was 9.6 months (95% CI 5.5-15.0) with tislelizumab plus chemotherapy versus 5.0 months (95% CI 4.1-6.9) with placebo plus chemotherapy (HR 0.45, 95% CI 0.26-0.78). ORR was 74.4% (95% CI 57.9%-87.0%) with tislelizumab plus chemotherapy versus 50.8% (95% CI 37.5%-64.1%) with placebo plus chemotherapy (Supplementary Table S10).

In the concordance analysis of PD-L1 TAP score at a 5% cutoff versus TC score at a 1% cutoff, positive percentage agreement was 86% (258/299 [95% CI 82%-90%]), negative percentage agreement was 59% (140/238 [95% CI 53%-65%]), and overall percentage agreement was 74% (398/537 [95% CI 71%-78%]).^1^

## Supplementary Discussion

**PD-L1 TAP score ≥5% by TC score subgroup analysis**

Exploratory analysis of TC score subgroups within the PD-L1 TAP score ≥5% population demonstrated consistent efficacy benefits with tislelizumab plus chemotherapy in both TC score ≥1% and TC score <1% subgroups, with substantial improvements in OS, PFS, and ORR observed regardless of TC score status (Supplementary Table S10). The overall percentage agreement between TAP score ≥5% and TC score ≥1% was 74%. As TAP score evaluates PD-L1 expression on both tumor cells and tumor-associated immune cells whereas TC score evaluates only tumor cells,^1^ these methodological differences may account for the moderate concordance. This has clinical relevance given that nivolumab, another PD-1 inhibitor approved for ESCC, has a TC score ≥1% threshold in the summary of product characteristics for the European Union.^2^ The efficacy observed in both TC subgroups within the TAP ≥5% population supports the use of TAP score as a biomarker for tislelizumab treatment selection in this indication.

# Supplementary References

1. Moehler M, Yoon HH, Wagner DC, et al. Concordance between the PD-L1 Tumor Area Positivity score and Combined Positive Score for gastric or esophageal cancers treated with tislelizumab. *Mod Pathol.* 2025;38(9):100793.

2. OPDIVO® (nivolumab) injection, for intravenous use [summary of product characteristics]. Available at. https://www.ema.europa.eu/en/documents/product-information/opdivo-epar-product-information_en.pdf. [Accessed 1 February 1, 2026].

# Supplementary Table S1. Institutional review board or independent ethics committees

| **Site No.** | **Name/address of ethics committee** | **Approval number** |
| --- | --- | --- |
| 61028  61052 | St. Vincent's Hospital Melbourne Human Research Ethics Committee, Melbourne, Australia | HREC 042/19 |
| 32001  32011  32013  32015  32017  32018 | Ethische Commissie Onderzoek, Leuven, Belgium | EudraCT No.: 2018-000587-28 |
| 86001 | Beijing Cancer Hospital, Beijing, China | 2018YW37 |
| 86006 | Henan Cancer Hospital – Oncology, Zhengzhou, China | 2018109 |
| 86007 | Jiangsu Province Hospital, Nanjing, China | 2018-MD-173;  2018-MD-173.A1 |
| 86009 | Peking Union Medical College, Beijing, China | KS2019018 |
| 86014 | The First Affiliated Hospital, Hangzhou, China | 2018伦审第(155)号 |
| 86018 | Subei People's Hospital, Yangzhou, China | 2018025-1 |
| 86020 | The Affiliated Hospital of Xuzhou, Xuzhou, China | XYFY2018-YL056-01 |
| 86022 | Harbin Medical University Cancer Hospital, Xuzhou, China | 2018-59 |
| 86029 | Anhui Provincial Hospital – Oncology, Anhui, China | 2018伦审第152号;  2018伦审第168号 |
| 86031 | The Second Hospital of Anhui, Anhui, China | YW2018-032(F1)]号 |
| 86033 | Fudan University Cancer Hospital, Shanghai, China | 1904199-9 |
| 86036 | Fujian Provincial Cancer Hospital, Fuzhou, China | 2018-035-01 |
| 86037 | The Second Affiliated Hospital, Nanchang, China | 药临审[2019]第(1)  号 |
| 86038 | Sir Run Run Shaw Hospital, Zhejiang, China | 20181106-3 |
| 86042 | Chinese PLA General Hospital, Beijing, China | C2021-040-01 |
| 86048 | Tianjin Cancer Hospital – Gastroenterology and Hepatology, Tianjin, China | E2018207 |
| 86049 | Hunan Cancer Hospital – GCP Office, Hunan, China | 2018年药审[158]号;  2018年快审[265]号 |
| 86053 | The First Affiliated Hospital, Weihui, China | [2018]伦药审字(35)  号 |
| 86054 | The First Affiliated Hospital, Fuzhou, China | LY2018-010-01;  LY2018-010-02 |
| 86056  86132 | The First Affiliated Hospital, Guangzhou, China | 086056  2018-046-01  2018-046-02  086132  ZYYEC[2018]012 |
| 86068 | The First Affiliated Hospital, Xiamen, China | [2018]药伦审字  (022)号 |
| 86055 | Hubei Cancer Hospital, Hubei, China | [2018]第25号 |
| 86057 | Xiangyang Central Hospital, Hubei, China | XYSZXYY-LL-PJ-2018-016 |
| 86058 | Shandong Linyi Tumor Hospital, Linyi, China | XY1824 |
| 86059 | Cancer Hospital of Shantou University, Shantou, China | 2018009 |
| 86066 | Shanxi Provincial People’s Hospital, Taiyuan, China | (2018)省医药伦审字  第30号 |
| 86075 | Liaoning Cancer Hospital & Institute, Shenyang, China | 20180942 |
| 86083 | Nantong Tumor Hospital, Nantong, China | 通肿伦审药试[2018-  013]号 |
| 86089 | Hainan General Hospital, Hainan, China | 医伦[2018]第71号 |
| 86101 | Zhejiang Cancer Hospital, Hangzhou, China | IRB-[2018]236号;  IRB-[2018]289号 |
| 86102 | Shandong Cancer Hospital, Jinan, China | SD2LEC2018-069-01 |
| 86103 | First Affiliated Hospital of Zhengzhou University, Zhengzhou, China | 药-2018-83 |
| 86108 | Guangxi Medical University Affiliated Cancer Hospital, Nanning, China | CS2018(42) |
| 86110 | The Sixth Affiliated Hospital, Guangzhou, China | 意见函2018-107;  2018ZSLYEC-080 |
| 86113 | Beijing Friendship Hospital, Beijing, China | 2018-P1-药017-01 |
| 86115 | Anhui Provincial Cancer Hospital, Hefei, China | 2018-伦理审查-24 |
| 86117 | The First People's Hospital of Changzhou, Changzhou, China | (2018)药第22号 |
| 86133 | The People’s Library Arm No. 307 Hospital, Beijing, China | 2018-5-24-1;  2018-5-24-2 |
| 86134 | Weifang People's Hospital, Weifang, China | 2018-020-01 |
| 86135 | Guangdong Province Traditional Chinese Medical Hospital, Shenzhen, China | 广东省中医院伦理  委员会AF2018-091-01 |
| 86139 | Quanzhou First Hospital, Quanzhou, China | 泉一伦[2018]25号；  泉一伦[2018]29号 |
| 86142 | Zhongshan Hospital Xiamen University, Zhongshan, China | XMZSIRB2018第031  号; 2018伦审第(17)  号 |
| 86157 | Wuxi Fourth People's Hospital, Wuxi, China | 2018-019-001 |
| 420004 | Fakultni nemocnice Olomouc, Olomouc, Czech Republic | EudraCT No.: 2018-000587-28 |
| 420011 | Fakultni Thomayerova nemocnice, Prague, Czech Republic | EudraCT No.: 2018-000587-28 |
| 33011  33030  33035  33036  33038  33039  33042  33050  33057  33059 | Comité de Protection des Perso, France | EudraCT No.: 2018-000587-28 |
| 49011  49012  49021  49031 | Ethikkommission der Landesärzt, Stuttgart, Germany | EudraCT No.: 2018-000587-28 |
| 39029 | Comitato Etico Di Area Vasta R, Modena, Italy | EudraCT No.: 2018-000587-28 |
| 39035 | Comitato Etico Degli Irccs Ist, Italy | EudraCT No.: 2018-000587-28 |
| 39054 | Azienda Ospedaliera Di Padova, Padova, Italy | EudraCT No.: 2018-000587-28 |
| 39056 | Comitato Etico Regionale (C.E.R), Bologna, Italy | EudraCT No.: 2018-000587-28 |
| 39063 | Comitato Etico IRCCS Pascale, Napoli, Italy | EudraCT No.: 2018-000587-28 |
| 81001 | Osaka University Hospital, Osaka, Japan | File No.: No. 180047-B |
| 81003 | Saitama Cancer Center, Saitama, Japan | File No.: AA019-04 |
| 81005 | Kyushu Medical Center, Kyushu, Japan | File No.: 19C10 |
| 81006 | Kumamoto University Hospital, Kumamoto, Japan | File No.: 2019011 |
| 81009  81015 | National Cancer Center Hospital, Chuo City, Tokyo, Japan | File No.: K0846 |
| 81016 | Kanagawa Cancer Center, Yokohama, Japan | File No.: 290 |
| 81017 | Akita University Hospital, Akita, Japan | File No.: 19008 |
| 81021 | University Hospital, Kyoto Prefectural University of Medicine, Kyoto, Japan | File No.: 2019-012 |
| 81026 | Hiroshima University Hospital, Hiroshima, Japan | File No.: 00951 |
| 81029 | Kyoto University Hospital, Kyoto, Japan | File No.: 2095 |
| 81030 | National Hospital Organization, Japan | File No.: 527 |
| 81031 | Osaka International Cancer Institute, Osaka, Japan | File No.: A-2019-05-0367 |
| 81033 | Kagawa University Hospital, Miki, Japan | File No.: 20191000117101 |
| 81035 | Hyogo Cancer Center, Akashi, Japan | File No.: HCC255 |
| 81056 | Kansai Rosai Hospital, Amagasaki, Japan | File No.: 19A011a |
| 81062 | Niigata Cancer Center Hospital, Niigata, Japan | File No.: NA, IRB approval date 11Oct2019 |
| 82012 | Irb Of Dongsan Hospital, Daegu, Republic of Korea | IRB file number: DSMC2019-02-015  Document number:  DSMC201902015-HE002 |
| 82015 | Gachon University Gil Medical, Incheon, Republic of Korea | IRB No.: GFIRB2019-101  Document No.:  GilHos IRB-2019-0748 |
| 82016 | Korea University Guro Hospital, Seoul, Republic of Korea | IRB No.: 2019GR0120  Document No.:  K2018-2180-001 |
| 82019 | The Catholic University of Korea, Seoul, Republic of Korea | Study No.: KC19MDGT0133  Receipt No.:  2019-0347-0001  Document No.:  KIRB-Full 20190305-010 |
| 82023 | CHA University – Bundang Medical Center, Seongnam, Republic of Korea | IRB File No.: CHAMC 2019-02-006-001  Document No.:  CHAMC201902006-HE001 |
| 82024 | SMG-SNU Boramae Medical Center, Republic of Korea | IRB No.: 20190425 / 30 - 2019 - 33 /053 |
| 82031 | Seoul National University Bund, Republic of Korea | IRB No.: B-1902 /522-007 |
| 82032 | Asan Medical Center, Republic of Korea | Study No.:  2018-1497  Receipt No.:  S2018-2164-0001 |
| 82034 | Chonnam National University, Gwangju, Republic of Korea | IRB No.: HTMP-2019-028 |
| 82035 | Samsung Medical Center, Republic of Korea | IRB File No.: SMC  2019-01-164-002  Document No.:  SMC201901164002-HE001 |
| 48009  48013  48019  48030  48041 | Komisja Bioetyczna przy NIO, Poland | EudraCT No.: 2018-000587-28 |
| 40002  40004  40007  40012  40013  40014 | Comisia de Bioetica, Romania | Reg. no. 4 S / 22 .01 .2019 |
| 7020 | Arkhangelsk Regional Clinical, Russian Federation | Minutes No. 801  Meeting of the Ethics Committee of the Arkhangelsk Clinical  Oncological Dispensary |
| 7025 | Leningrad Regional Oncology Dispensary, Leningrad, Russian Federation | Meeting of the Local Ethics Committee at the GBUZ LOKOD No. 04-01 /2019 |
| 7027 | Ivanovo Regional Oncological Dispensary, Ivanovo, Russian Federation | Minutes No. 05 /19 of the meeting of the Committee on Ethical Matters  at the OBUZ |
| 7044 | The First Saint-Petersburg Sta, Russian Federation | Minutes No. 218  of the Meeting of the Local Ethics Committee  at FGBOU VO |
| 7045 | Petrov Research Institute of O, Russian Federation | No. 3 /50 from Minutes No. 8 of the Regular meeting of the Ethics Committee  at the FGBU |
| 7047 | Rostov Research Institute of O, Russian Federation | MINUTES No. 17  of the meeting of the Ethics Board at FGBU |
| 7048 | Orenburg Regional Clinical Oncology Center, Orenburg, Russian Federation | Minutes No.92 of the meeting of the Local Ethics Committee  at the GBUZ |
| 34017  34018  34026  34028  34033  34034  34039 | CEIm Regional Madrid, Madrid, Spain | EudraCT No.: 2018-000587-28  Ref: 07 /674294 .9 /19 |
| 886004  886009 | Chi Mei Medical Center, Taiwan | 886004: IRB Seiial No.: 10804—L03  886009: IRB Seiial No.: 10804—005 |
| 886012 | Kaohsiung Veterans General Hospital, Taiwan | IRB No.: VGHKS19-CT5-14 |
| 886044 | Chang Gung Medical Foundation, Taiwan | IRB No.: 201802166A0 |
| 886048 | China Medical University Hospital, Taiwan | CMUH REC No.: CMUH108-REC2-078 |
| 44011  44012  44015  44034  44045 | East of England – Essex Research, United Kingdom | IRAS project ID: 252786  EudraCT number: 2018-000587-28 |
| 1159  1161  1235 | Advarra, United States | 1159: SSU00082455  1161: SSU00089440  1235: SSU00080807 |

## Supplementary Table S2. Baseline demographic and clinical characteristics in patients with PD-L1 TAP score ≥5%

|  | **Tislelizumab plus chemotherapy (*n* = 172)** | **Placebo plus chemotherapy (*n* = 186)** |
| --- | --- | --- |
| Age, years |  |  |
| Median (IQR) | 62.0 (56.5-67.0) | 64.0 (58.0-70.0) |
| <65, *n* (%) | 100 (58.1) | 98 (52.7) |
| ≥65, *n* (%) | 72 (41.9) | 88 (47.3) |
| Sex, *n* (%) |  |  |
| Male | 141 (82.0) | 163 (87.6) |
| Female | 31 (18.0) | 23 (12.4) |
| Geographical region, *n* (%) |  |  |
| Asia | 133 (77.3) | 147 (79.0) |
| Japan | 17 (9.9) | 16 (8.6) |
| Rest of world | 39 (22.7) | 39 (21.0) |
| Race, *n* (%) |  |  |
| American Indian or Alaska native | 0 (0.0) | 1 (0.5) |
| Asian | 133 (77.3) | 147 (79.0) |
| White | 39 (22.7) | 36 (19.4) |
| Not reported or unknown | 0 (0.0) | 2 (1.1) |
| Median BMI, kg/m^2^ (IQR) | 21.2 (19.4-23.1) | 21.2 (19.0-24.2) |
| ECOG performance status, *n* (%) |  |  |
| 0 | 50 (29.1) | 57 (30.6) |
| 1 | 122 (70.9) | 129 (69.4) |
| Smoking status, *n* (%) |  |  |
| Never | 35 (20.3) | 45 (24.2) |
| Current or former | 131 (76.2) | 132 (71.0) |
| Missing | 6 (3.5) | 9 (4.8) |
| Disease status at study entry, *n* (%) |  |  |
| Locally advanced | 25 (14.5) | 20 (10.8) |
| Metastatic | 147 (85.5) | 166 (89.2) |
| Number of metastatic sites at study entry, *n* (%) |  |  |
| 0 | 25 (14.5) | 20 (10.8) |
| 1 | 75 (43.6) | 87 (46.8) |
| 2 | 46 (26.7) | 45 (24.2) |
| ≥3 | 26 (15.1) | 34 (18.3) |
| Histologic type, *n* (%) |  |  |
| Squamous cell carcinoma | 172 (100.0) | 186 (100.0) |
| Previous definitive therapy, *n* (%) | 68 (39.5) | 73 (39.2) |
| Definitive surgery with/without adjuvant/neo-adjuvant treatment | 53 (30.8) | 57 (30.6) |
| Definitive radiotherapy with/without chemotherapy | 18 (10.5) | 19 (10.2) |
| Prior anti-cancer systemic therapy, *n* (%) | 46 (26.7) | 59 (31.7) |

Data cutoff: 22 August 2024.

BMI, body mass index; ECOG, Eastern Cooperative Oncology Group; IQR, interquartile range; ITT, intent-to treat; PD-L1, programmed death-ligand 1; TAP, Tumor Area Positivity.

## Supplementary Table S3. Treatment exposure in patients with PD-L1 TAP score ≥5% at 3- year minimum follow-up

|  | **Tislelizumab plus chemotherapy (*n* = 171)** | **Placebo plus chemotherapy (*n* = 185)** |
| --- | --- | --- |
| Duration of treatment (month),^a^ median (IQR) | 6.9 (4.2-15.2) | 4.9 (2.8-8.2) |
| Duration of treatment, *n* (%) |  |  |
| <1 month | 10 (5.8) | 7 (3.8) |
| ≥1 to <3 months | 18 (10.5) | 47 (25.4) |
| ≥3 to <6 months | 47 (27.5) | 57 (30.8) |
| ≥6 to <12 months | 46 (26.9) | 53 (28.6) |
| ≥12 to <18 months | 16 (9.4) | 8 (4.3) |
| ≥18 to <24 months | 6 (3.5) | 7 (3.8) |
| ≥24 months | 28 (16.4) | 6 (3.2) |
| Number of cycles received, median (IQR) | 9.0 (6.0-19.0) | 7.0 (4.0-11.0) |
| Number of cycles received, *n* (%) |  |  |
| 1-3 | 24 (14.0) | 45 (24.3) |
| 4-6 | 26 (15.2) | 45 (24.3) |
| 7-9 | 43 (25.1) | 34 (18.4) |
| 10-12 | 18 (10.5) | 26 (14.1) |
| 13-18 | 17 (9.9) | 18 (9.7) |
| 19-24 | 9 (5.3) | 6 (3.2) |
| 25-36 | 15 (8.8) | 8 (4.3) |
| >36 | 19 (11.1) | 3 (1.6) |
| Cumulative total dose (mg) per patient,^b^ median (IQR) | 1800.0 (1200.0-3800.0) | 1400.0 (800.0-2200.0) |
| Actual dose intensity (mg/cycle) per patient,^c^ median (IQR) | 193.5 (175.0-199.3) | 192.4 (182.6-200.0) |
| Relative dose intensity (%) per patient,^d^ median (IQR) | 96.8 (87.5-99.6) | 96.2 (91.3-100.0) |
| Number of patients treated beyond investigator-assessed radiological progression, *n* (%) | 29 (17.0) | 24 (13.0) |
| Patients with any dose modification, *n* (%) | 103 (60.2) | 97 (52.4) |
| Dose delay | 103 (60.2) | 97 (52.4) |
| Adverse event | 77 (45.0) | 56 (30.3) |
| Other | 60 (35.1) | 54 (29.2) |
| Related to COVID-19 | 29 (17.0) | 24 (13.0) |
| Infusion interruption/infusion rate decrease | 3 (1.8) | 0 (0.0) |
| Adverse event | 1 (0.6) | 0 (0.0) |
| Other | 2 (1.2) | 0 (0.0) |
| Related to COVID-19 | 0 (0.0) | 0 (0.0) |

Data cutoff: 24 November 2023. Percentages were based on the total number of patients in the relevant treatment group.

IQR, interquartile range; PD-L1, programmed death-ligand 1; TAP, Tumor Area Positivity.

^a^Duration of treatment = (min (last dose date + 20, death date, cutoff date) − first dose date + 1) / 30.4375, if patients discontinued from treatment; duration of treatment = (cutoff date – first dose date + 1) / 30.4375, if patients with ongoing treatment.

^b^Cumulative total dose per patient is the sum of actual received doses of all visits per patient.

^c^Actual dose intensity is 21*cumulative total dose (mg) / (last dose date up to cutoff date + 21 – first dose date).

^d^Relative dose intensity is actual dose intensity / (200 mg/cycle).

## Supplementary Table S4. Treatment exposure in patients with PD-L1 TAP score ≥5% at 45.2-months minimum follow-up

|  | **Tislelizumab plus chemotherapy (*n* = 171)** | **Placebo plus chemotherapy (*n* = 185)** |
| --- | --- | --- |
| Duration of treatment (month),^a^ median (IQR) | 6.9 (4.2-15.2) | 4.9 (2.8-8.2) |
| Duration of treatment, *n* (%) |  |  |
| <1 month | 10 (5.8) | 7 (3.8) |
| ≥1 to <3 months | 18 (10.5) | 47 (25.4) |
| ≥3 to <6 months | 47 (27.5) | 57 (30.8) |
| ≥6 to <12 months | 46 (26.9) | 53 (28.6) |
| ≥12 to <18 months | 16 (9.4) | 8 (4.3) |
| ≥18 to <24 months | 6 (3.5) | 7 (3.8) |
| ≥24 months | 28 (16.4) | 6 (3.2) |
| Number of cycles received, median (IQR) | 9.0 (6.0-19.0) | 7.0 (4.0-11.0) |
| Number of cycles received, *n* (%) |  |  |
| 1-3 | 24 (14.0) | 45 (24.3) |
| 4-6 | 26 (15.2) | 45 (24.3) |
| 7-9 | 43 (25.1) | 34 (18.4) |
| 10-12 | 18 (10.5) | 26 (14.1) |
| 13-18 | 17 (9.9) | 18 (9.7) |
| 19-24 | 9 (5.3) | 6 (3.2) |
| 25-36 | 15 (8.8) | 8 (4.3) |
| >36 | 19 (11.1) | 3 (1.6) |
| Cumulative total dose (mg) per patient,^b^ median (IQR) | 1800.0 (1200.0-3800.0) | 1400.0 (800.0-2200.0) |
| Actual dose intensity (mg/cycle) per patient,^c^ median (IQR) | 193.6 (175.0-199.3) | 192.4 (182.6-200.0) |
| Relative dose intensity (%) per patient,^d^ median (IQR) | 96.8 (87.5-99.6) | 96.2 (91.3-100.0) |
| Number of patients treated beyond investigator-assessed radiological progression, *n* (%) | 29 (17.0) | 24 (13.0) |
| Patients with any dose modification, *n* (%) | 103 (60.2) | 97 (52.4) |
| Dose delay | 103 (60.2) | 97 (52.4) |
| Adverse event | 77 (45.0) | 56 (30.3) |
| Other | 60 (35.1) | 54 (29.2) |
| Related to COVID-19 | 29 (17.0) | 24 (13.0) |
| Infusion interruption/infusion rate decrease | 3 (1.8) | 0 (0.0) |
| Adverse event | 1 (0.6) | 0 (0.0) |
| Other | 2 (1.2) | 0 (0.0) |
| Related to COVID-19 | 0 (0.0) | 0 (0.0) |

Data cutoff: 22 August 2024. Percentages were based on the total number of patients in the relevant treatment group.

IQR, interquartile range; PD-L1, programmed death-ligand 1; TAP, Tumor Area Positivity.

^a^Duration of treatment = (min (last dose date + 20, death date, cutoff date) – first dose date + 1) / 30.4375, if patients discontinued from treatment; duration of treatment = (cutoff date – first dose date + 1) / 30.4375, if patients with ongoing treatment.

^b^Cumulative total dose per patient is the sum of actual received doses of all visits per patient.

^c^Actual dose intensity is 21*cumulative total dose (mg) / (last dose date up to cutoff date + 21 – first dose date).

^d^Relative dose intensity is actual dose intensity / (200 mg/cycle).

## Supplementary Table S5. Exposure to chemotherapy doublet A (platinum plus 5FU) in patients with PD-L1 TAP score ≥5% at 45.2-months minimum follow-up

|  | **Platinum plus 5FU** | | | |
| --- | --- | --- | --- | --- |
|  | **Tislelizumab plus chemotherapy (*n* = 48)** | | **Placebo plus chemotherapy (*n* = 50)** | |
|  | **Platinum  (m = 48)** | **5-FU**  **(m = 48)** | **Platinum**  **(m = 50)** | **5-FU (m = 49)** |
| Duration of treatment (month),^a^ median (IQR) | 4.3 (3.7-5.1) | 4.4 (3.7-7.1) | 4.2 (2.5-4.8) | 4.8 (3.3-6.9) |
| Duration of treatment, *n* (%) |  |  |  |  |
| <1 month | 4 (8.3) | 4 (8.3) | 3 (6.0) | 2 (4.1) |
| ≥1 to <3 months | 6 (12.5) | 6 (12.5) | 10 (20.0) | 9 (18.4) |
| ≥3 to <6 months | 30 (62.5) | 22 (45.8) | 31 (62.0) | 22 (44.9) |
| ≥6 to <12 months | 8 (16.7) | 9 (18.8) | 5 (10.0) | 13 (26.5) |
| ≥12 to ≤18 months | 0 (0.0) | 4 (8.3) | 0 (0.0) | 1 (2.0) |
| >18 months | 0 (0.0) | 3 (6.3) | 1 (2.0) | 2 (4.1) |
| Number of cycles received, median (IQR) | 6.0 (4.5-6.0) | 6.0 (5.0-9.0) | 6.0 (3.0-6.0) | 6.0 (4.0-8.0) |
| Number of cycles received, *n* (%) |  |  |  |  |
| 1-3 | 9 (18.8) | 9 (18.8) | 14 (28.0) | 12 (24.5) |
| 4-6 | 28 (58.3) | 21 (43.8) | 26 (52.0) | 15 (30.6) |
| 7-9 | 7 (14.6) | 8 (16.7) | 8 (16.0) | 11 (22.4) |
| 10-12 | 4 (8.3) | 2 (4.2) | 1 (2.0) | 6 (12.2) |
| 13-18 | 0 (0.0) | 2 (4.2) | 0 (0.0) | 3 (6.1) |
| >18 | 0 (0.0) | 6 (12.5) | 1 (2.0) | 2 (4.1) |
| Cumulative total dose (mg/m^2^) per patient,^b^ median (IQR) | NA (NA-NA) | 22485.6  (16376.3-34952.4) | NA (NA-NA) | 22127.5  (11706.0-27983.0) |
| Actual dose intensity (mg/m^2^/cycle) per patient,^c^ median (IQR) | NA (NA-NA) | 3450.1  (2941.5-3717.0) | NA (NA-NA) | 3323.6  (2576.2-3684.1) |
| Relative dose intensity (%) per patient,^d^ median (IQR) | 91.1 (75.9-98.2) | 86.3 (74.3-95.9) | 88.2 (71.6-97.4) | 87.1 (67.5-96.9) |
| Number of patients treated beyond investigator-assessed radiological progression, *n* (%) | 1 (2.1) | 2 (4.2) | 1 (2.0) | 4 (8.2) |
| Patients with any dose modification, *n* (%) | 31 (64.6) | 35 (72.9) | 36 (72.0) | 38 (77.6) |
| Dose delay | 28 (58.3) | 30 (62.5) | 28 (56.0) | 30 (61.2) |
| Adverse event | 24 (50.0) | 26 (54.2) | 22 (44.0) | 22 (44.9) |
| Other | 9 (18.8) | 11 (22.9) | 10 (20.0) | 13 (26.5) |
| Related to COVID-19 | 2 (4.2) | 2 (4.2) | 3 (6.0) | 6 (12.2) |
| Infusion interruption/ infusion rate decrease | 1 (2.1) | 13 (27.1) | 2 (4.0) | 10 (20.4) |
| Adverse event | 0 (0.0) | 4 (8.3) | 1 (2.0) | 3 (6.1) |
| Other | 1 (2.1) | 12 (25.0) | 1 (2.0) | 8 (16.3) |
| Related to COVID-19 | 0 (0.0) | 0 (0.0) | 0 (0.0) | 0 (0.0) |
| Dose reduction | 16 (33.3) | 14 (29.2) | 25 (50.0) | 22 (44.9) |
| Adverse event | 16 (33.3) | 14 (29.2) | 23 (46.0) | 22 (44.9) |
| Other | 0 (0.0) | 1 (2.1) | 2 (4.0) | 0 (0.0) |
| Related to COVID-19 | 0 (0.0) | 0 (0.0) | 0 (0.0) | 0 (0.0) |

Data cutoff: 22 August 2024. Percentages were based on m, where m was the number of patients taking at least one dose of chemotherapy agent under corresponding column.

Chemotherapy doublet A: Cisplatin (60-80 mg/m^2^ Q3W IV) or oxaliplatin (130 mg/m^2^ Q3W IV) and 5-Fluorouracil (750-800 mg/m^2^ Q3W IV).
5FU, 5-Fluorouracil; CRF, case report form; IQR, interquartile range; IV, intravenous; NA, not available; PD-L1, programmed death-ligand 1; Q3W, every 3 weeks; TAP, Tumor Area Positivity.

^a^Duration of treatment for patients discontinued from treatment: For cisplatin or oxaliplatin on a Q3W schedule, duration of treatment = (min (last dose date + 20, death date, cutoff date) – first dose date + 1) / 30.4375; For 5-FU, duration of treatment = (min (last dose date + 16, death date, cutoff date) – first dose date + 1) /30.4375. Duration of treatment for patients with ongoing treatment = (cutoff date – first dose date + 1) /30.4375.

^b^Cumulative total dose per patient is the sum of actual received doses of all visits per patient for each study drug.

^c^Actual dose intensity is cumulative total dose (mg/m^2^) / (last dose date up to cutoff date + 21 – first dose date) /21 for oxaliplatin; max ((last dose date up to cutoff date + 21 – first dose date) /21, number of cycles in last CRF page) for cisplatin; max ((last dose date up to cutoff date + 17 – first dose date) /21, number of cycles in last CRF page) for 5-FU.

^d^Relative dose intensity is actual dose intensity/planned dose intensity. The planned dose intensity is the total planned dose in first cycle.

## Supplementary Table S6. Exposure to chemotherapy doublet B (platinum plus capecitabine) in patients with PD-L1 TAP score ≥5% at 45.2-months minimum follow-up

|  | **Platinum plus capecitabine** | | | |
| --- | --- | --- | --- | --- |
|  | **Tislelizumab plus chemotherapy (*n* = 20)** | | **Placebo plus chemotherapy (*n* = 27)** | |
|  | **Platinum  (m = 20)** | **Capecitabine**  **(m = 20)** | **Platinum**  **(m = 27)** | **Capecitabine**  **(m = 27)** |
| Duration of treatment (month),^a^ median (IQR) | 4.9 (4.2-6.2) | 10.3 (6.1-17.7) | 3.1 (2.5-4.2) | 3.1 (2.5-5.6) |
| Duration of treatment,  *n* (%) |  |  |  |  |
| <1 month | 0 (0.0) | 0 (0.0) | 0 (0.0) | 2 (7.4) |
| ≥1 to <3 months | 0 (0.0) | 0 (0.0) | 13 (48.1) | 9 (33.3) |
| ≥3 to <6 months | 14 (70.0) | 4 (20.0) | 13 (48.1) | 11 (40.7) |
| ≥6 to <12 months | 6 (30.0) | 9 (45.0) | 1 (3.7) | 2 (7.4) |
| ≥12 to ≤18 months | 0 (0.0) | 2 (10.0) | 0 (0.0) | 2 (7.4) |
| >18 months | 0 (0.0) | 5 (25.0) | 0 (0.0) | 1 (3.7) |
| Number of cycles received, median (IQR) | 6.0 (6.0-8.5) | 13.5 (7.5-21.0) | 4.0 (3.0-6.0) | 4.0 (3.0-7.0) |
| Number of cycles received, *n* (%) |  |  |  |  |
| 1-3 | 0 (0.0) | 0 (0.0) | 9 (33.3) | 9 (33.3) |
| 4-6 | 12 (60.0) | 3 (15.0) | 17 (63.0) | 11 (40.7) |
| 7-9 | 6 (30.0) | 4 (20.0) | 0 (0.0) | 3 (11.1) |
| 10-12 | 1 (5.0) | 3 (15.0) | 0 (0.0) | 0 (0.0) |
| 13-18 | 1 (5.0) | 3 (15.0) | 1 (3.7) | 2 (7.4) |
| >18 | 0 (0.0) | 7 (35.0) | 0 (0.0) | 2 (7.4) |
| Cumulative total dose (mg/m^2^) per patient,^b^ median (IQR) | NA (NA-NA) | 294089.2  (191350.5-531559.2) | NA (NA-NA) | 109986.4  (74933.6-170333.5) |
| Actual dose intensity (mg/m^2^/cycle) per patient,^c^ median (IQR) | NA (NA-NA) | 22681.2  (18576.5-24252.3) | NA (NA-NA) | 22164.8  (16024.5-24757.5) |
| Relative dose intensity (%) per patient,^d^ median (IQR) | 88.9 (79.1-94.5) | 81.0 (66.3-86.6) | 87.9 (77.9-97.9) | 79.2 (57.2-88.4) |
| Number of patients treated beyond investigator-assessed radiological progression, *n* (%) | 1 (5.0) | 9 (45.0) | 1 (3.7) | 3 (11.1) |
| Patients with any dose modification, *n* (%) | 17 (85.0) | 19 (95.0) | 19 (70.4) | 18 (66.7) |
| Dose delay | 16 (80.0) | 18 (90.0) | 17 (63.0) | 18 (66.7) |
| Adverse event | 15 (75.0) | 18 (90.0) | 15 (55.6) | 15 (55.6) |
| Other | 2 (10.0) | 7 (35.0) | 2 (7.4) | 5 (18.5) |
| Related to COVID-19 | 1 (5.0) | 2 (10.0) | 0 (0.0) | 1 (3.7) |
| Infusion interruption/ infusion rate decrease | 1 (5.0) | NA | 2 (7.4) | NA |
| Adverse event | 1 (5.0) | NA | 2 (7.4) | NA |
| Other | 0 (0.0) | NA | 0 (0.0) | NA |
| Related to COVID-19 | 0 (0.0) | NA | 0 (0.0) | NA |
| Dose reduction | 6 (30.0) | 10 (50.0) | 11 (40.7) | 8 (29.6) |
| Adverse event | 6 (30.0) | 10 (50.0) | 10 (37.0) | 7 (25.9) |
| Other | 0 (0.0) | 0 (0.0) | 1 (3.7) | 0 (0.0) |
| Related to COVID-19 | 0 (0.0) | 0 (0.0) | 0 (0.0) | 0 (0.0) |

Data cutoff: 22 August 2024. Percentages were based on m, where m was the number of patients taking at least one dose of chemotherapy agent under corresponding column.

Chemotherapy doublet B: Cisplatin (60-80 mg/m^2^ Q3W IV) or oxaliplatin (130 mg/m^2^ Q3W IV) and capecitabine (1000 mg/m^2^ twice a day.
CRF, case report form; IQR, interquartile range; IV, intravenous; NA, not available; PD-L1, programmed death-ligand 1; Q3W, every 3 weeks; TAP, Tumor Area Positivity.

^a^Duration of treatment for patients discontinued from treatment: For cisplatin or oxaliplatin on a Q3W schedule, duration of treatment = (min (last dose date + 20, death date, cutoff date) – first dose date + 1) / 30.4375; For capecitabine, duration of treatment = (min (last dose date + 7, death date, cutoff date) – first dose date + 1) /30.4375. Duration of treatment for patients with ongoing treatment = (cutoff date – first dose date + 1) / 30.4375.

^b^Cumulative total dose per patient is the sum of actual received doses of all visits per patient for each study drug.

^c^Actual dose intensity is cumulative total dose (mg/m^2^) / (last dose date up to cutoff date + 21 – first dose date) /21 for oxaliplatin; max ((last dose date up to cutoff date + 21 – first dose date) /21, number of cycles in last CRF page) for cisplatin; max ((last dose date up to cutoff date + 8 – first dose date) /21, number of cycles in last CRF page) for capecitabine.

^d^Relative dose intensity is actual dose intensity/planned dose intensity. The planned dose intensity is the total planned dose in first cycle.

**Supplementary Table S7. Exposure to chemotherapy doublet C (platinum plus paclitaxel) in patients with PD-L1 TAP score ≥5% at 45.2-months minimum follow-up**

|  | **Platinum plus paclitaxel** | | | |
| --- | --- | --- | --- | --- |
|  | **Tislelizumab plus chemotherapy (*n* = 103)** | | **Placebo plus chemotherapy (*n* = 108)** | |
|  | **Platinum  (m = 103)** | **Paclitaxel**  **(m = 103)** | **Platinum**  **(m = 108)** | **Paclitaxel**  **(m = 108)** |
| Duration of treatment (month),^a^ median (IQR) | 4.1 (3.5-4.4) | 4.4 (3.5-5.8) | 4.1 (2.7-4.3) | 4.1 (2.7-5.5) |
| Duration of treatment,  *n* (%) |  |  |  |  |
| <1 month | 4 (3.9) | 5 (4.9) | 5 (4.6) | 5 (4.6) |
| ≥1 to <3 months | 17 (16.5) | 16 (15.5) | 30 (27.8) | 27 (25.0) |
| ≥3 to <6 months | 75 (72.8) | 57 (55.3) | 73 (67.6) | 60 (55.6) |
| ≥6 to <12 months | 7 (6.8) | 17 (16.5) | 0 (0.0) | 14 (13.0) |
| ≥12 to ≤18 months | 0 (0.0) | 5 (4.9) | 0 (0.0) | 1 (0.9) |
| >18 months | 0 (0.0) | 3 (2.9) | 0 (0.0) | 1 (0.9) |
| Number of cycles received, median (IQR) | 6.0 (5.0-6.0) | 6.0 (5.0-8.0) | 6.0 (4.0-6.0) | 6.0 (4.0-8.0) |
| Number of cycles received, *n* (%) |  |  |  |  |
| 1-3 | 14 (13.6) | 13 (12.6) | 26 (24.1) | 25 (23.1) |
| 4-6 | 85 (82.5) | 53 (51.5) | 78 (72.2) | 50 (46.3) |
| 7-9 | 1 (1.0) | 20 (19.4) | 4 (3.7) | 22 (20.4) |
| 10-12 | 3 (2.9) | 7 (6.8) | 0 (0.0) | 9 (8.3) |
| 13-18 | 0 (0.0) | 5 (4.9) | 0 (0.0) | 1 (0.9) |
| >18 | 0 (0.0) | 5 (4.9) | 0 (0.0) | 1 (0.9) |
| Cumulative total dose (mg/m^2^) per patient,^b^ median (IQR) | NA (NA-NA) | 1000.5  (715.4-1225.0) | NA (NA-NA) | 932.0  (575.0-1077.8) |
| Actual dose intensity (mg/m^2^/cycle) per patient,^c^ median (IQR) | NA (NA-NA) | 152.2  (132.4-172.9) | NA (NA-NA) | 160.2 (136.2-173.6) |
| Relative dose intensity (%) per patient,^d^ median (IQR) | 97.2 (88.0-99.7) | 87.0 (75.6-98.8) | 97.4 (91.4-99.7) | 91.6 (77.9-99.2) |
| Number of patients treated beyond investigator-assessed radiological progression, *n* (%) | 9 (8.7) | 13 (12.6) | 5 (4.6) | 8 (7.4) |
| Patients with any dose modification, *n* (%) | 47 (45.6) | 72 (69.9) | 47 (43.5) | 69 (63.9) |
| Dose delay | 34 (33.0) | 44 (42.7) | 38 (35.2) | 43 (39.8) |
| Adverse event | 20 (19.4) | 29 (28.2) | 16 (14.8) | 19 (17.6) |
| Other | 18 (17.5) | 24 (23.3) | 23 (21.3) | 26 (24.1) |
| Related to COVID-19 | 5 (4.9) | 9 (8.7) | 7 (6.5) | 12 (11.1) |
| Infusion interruption/ infusion rate decrease | 5 (4.9) | 6 (5.8) | 1 (0.9) | 10 (9.3) |
| Adverse event | 4 (3.9) | 6 (5.8) | 1 (0.9) | 8 (7.4) |
| Other | 1 (1.0) | 0 (0.0) | 0 (0.0) | 2 (1.9) |
| Related to COVID-19 | 0 (0.0) | 0 (0.0) | 0 (0.0) | 0 (0.0) |
| Dose reduction | 17 (16.5) | 50 (48.5) | 17 (15.7) | 49 (45.4) |
| Adverse event | 16 (15.5) | 49 (47.6) | 17 (15.7) | 48 (44.4) |
| Other | 1 (1.0) | 1 (1.0) | 0 (0.0) | 3 (2.8) |
| Related to COVID-19 | 0 (0.0) | 0 (0.0) | 0 (0.0) | 0 (0.0) |

Data cutoff: 22 August 2024. Percentages were based on m. m number of patients taking at least one dose of chemotherapy agent under corresponding column.

Chemotherapy doublet C: Cisplatin (60-80 mg/m^2^ Q3W IV) or oxaliplatin (130 mg/m^2^ Q3W IV) and paclitaxel (175 mg/m^2^ Q3W IV).
CRF, case report form; IQR, interquartile range; IV, intravenous; NA, not available; PD-L1, programmed death-ligand 1; Q3W, every 3 weeks; TAP, Tumor Area Positivity.

^a^For oxaliplatin or paclitaxel on a Q3W schedule, duration of treatment = (min (last dose date + 20, death date, cutoff date) – first dose date + 1) / 30.4375; For cisplatin, duration of treatment = (min (last dose date + 20 (for Day 1 or Day 2 schedule) or 18 (for 3 days schedule), death date, cutoff date) – date of first dose + 1) /30.4375. Duration of treatment for patients with ongoing treatment = (cutoff date – first dose date + 1) /30.4375.

^b^Cumulative total dose per patient is the sum of actual received doses of all visits per patient.

^c^Actual dose intensity is cumulative total dose (mg/m^2^) / (last dose date up to cutoff date + 21 – first dose date) /21 for oxaliplatin or paclitaxel; max ((last dose date up to cutoff date + 21 (for Day 1 or Day 2 schedule) or 19 (for 3 days schedule) – first dose date) /21, number of cycles in last CRF page) for cisplatin.

^d^Relative dose intensity is actual dose intensity/planned dose intensity. The planned dose intensity is the total planned dose in first cycle.

## Supplementary Table S8. Response outcomes in patients with PD-L1 TAP score ≥5% at 3- year minimum follow-up

|  | **Tislelizumab plus chemotherapy (*n* = 172)** | **Placebo plus chemotherapy (*n* = 186)** |
| --- | --- | --- |
| Median OS, months (95% CI) | 19.1 (16.1-24.1) | 10.0 (8.6-11.9) |
| HR (95% CI)^a^ | 0.62 (0.49-0.79) |  |
| One-sided stratified  log-rank test *P* value^b,c^ | < 0.0001 |  |
| Median PFS, months (95% CI) | 8.2 (7.0-9.8) | 5.5 (4.3-6.4) |
| HR (95% CI)^a^ | 0.50 (0.39-0.65) |  |
| One-sided stratified  log-rank test *P* value^b^ | < 0.0001 |  |
| Objective response rate, *n* | 123 | 77 |
| % (95% CI)^d^ | 71.5 (64.1-78.1) | 41.4 (34.2-48.8) |
| Odds ratio, (95% CI)^e^ | 3.55 (2.27-5.53) |  |
| Two-sided CMH test *P* value^f^ | <0.0001 |  |
| ORR difference, % (95% CI)^e^ | 30.0 (20.2-39.7) |  |
| Best overall response, n (%) |  |  |
| Complete response | 12 (7.0) | 3 (1.6) |
| Partial response | 111 (64.5) | 74 (39.8) |
| Stable disease^g^ | 35 (20.3) | 78 (41.9) |
| Progressive disease | 6 (3.5) | 22 (11.8) |
| Could not be determined^h^ | 8 (4.7) | 9 (4.8) |
| Disease control rate, *n* | 158 | 155 |
| % (95% CI)^d^ | 91.9 (86.7-95.5) | 83.3 (77.2-88.4) |
| Odds ratio, (95% CI)^e^ | 2.22 (1.15-4.31) |  |
| Two-sided CMH test *P* value^f^ | 0.0157 |  |
| DCR difference, % (95% CI)^e^ | 8.5 (1.7-15.4) |  |

Data cutoff: 24 November 2023. Percentages were based on the total number of patients in the relevant treatment group. ORR is unconfirmed and is defined as proportion of number of patients with a PR or CR per RECIST v1.1 (i.e. ORR = CR + PR).

CI, confidence interval; CMH, Cochran-Mantel-Haenszel; CR, complete response; DCR, disease control rate; HR, hazard ratio; IRT, interactive response technology; ORR, objective response rate; OS, overall survival; PR, partial response; PD, progressive disease; PD-L1, programmed death-ligand 1; PFS, progression-free survival; RECIST, Response Evaluation Criteria in Solid Tumors; SD, stable disease; TAP, Tumor Area Positivity.

^a^Stratified HR (tislelizumab plus chemotherapy versus placebo plus chemotherapy) was based on Cox regression model including treatment arm as a covariate and stratified by pooled geographic region (Asia versus rest of world), prior definitive therapy (yes versus no), and investigator choice of chemotherapy (platinum with fluoropyrimidine versus platinum with paclitaxel) per IRT.

^b^One-sided *P* value was estimated from log rank test stratified by pooled geographic region (Asia versus rest of world), prior definitive therapy (yes versus no), and investigator choice of chemotherapy (platinum with fluoropyrimidine versus platinum with paclitaxel) per IRT.

^c^Presented for descriptive purpose only.

^d^Two-sided 95% CI was calculated using Clopper-Pearson method.
^e^Differences and odds ratios between arms were calculated using the CMH method, stratified by pooled geographic region (Asia versus rest of world), prior definitive therapy (yes versus no), and investigator choice of chemotherapy (platinum with fluoropyrimidine versus platinum with paclitaxel) per IRT.
^f^Two-sided CMH test was stratified by pooled geographic region, prior definitive therapy, and investigator choice of chemotherapy per IRT.

^g^SD includes SD and non-CR/non-PD.

^h^Included patients with no postbaseline response assessment (Not Assessable) or assessment as Not Evaluable (NE) per RECIST v1.1.

## Supplementary Table S9. Response outcomes in patients with PD-L1 TAP score ≥5% at 45.2-months minimum follow-up

|  | **Tislelizumab plus chemotherapy (*n* = 172)** | **Placebo plus chemotherapy (*n* = 186)** |
| --- | --- | --- |
| Objective response rate, *n* | 123 | 77 |
| % (95% CI)^a^ | 71.5 (64.1-78.1) | 41.4 (34.2-48.8) |
| Odds ratio, (95% CI)^b^ | 3.55 (2.27-5.53) | – |
| Two-sided CMH test *P* value^c^ | < 0.0001 | – |
| ORR difference, % (95% CI)^b^ | 30.0 (20.2-39.7) | – |
| Best overall response, n (%) |  |  |
| Complete response | 12 (7.0) | 3 (1.6) |
| Partial response | 111 (64.5) | 74 (39.8) |
| Stable disease^d^ | 35 (20.3) | 78 (41.9) |
| Progressive disease | 6 (3.5) | 22 (11.8) |
| Could not be determined^e^ | 8 (4.7) | 9 (4.8) |
| Disease control rate, *n* | 158 | 155 |
| % (95% CI)^a^ | 91.9 (86.7-95.5) | 83.3 (77.2-88.4) |
| Odds ratio, (95% CI)^b^ | 2.22 (1.15-4.31) |  |
| Two-sided CMH test *P* value^c^ | 0.0157 |  |
| DCR difference, % (95% CI)^b^ | 8.5 (1.7-15.4) |  |

Data cutoff: 22 August 2024. Percentages were based on the total number of patients in the relevant treatment group. ORR is unconfirmed and is defined as proportion of number of patients with a PR or CR per RECIST v1.1 (i.e. ORR = CR + PR).

CMH, Cochran-Mantel Haenszel; CR, complete response; DCR, disease control rate; IRT, interactive response technology; ORR, objective response rate; PR, partial response; PD, progressive disease; RECIST, Response Evaluation Criteria in Solid Tumors; SD, stable disease; TAP, Tumor Area Positivity.

^a^Two-sided 95% CI was calculated using Clopper-Pearson method.
^b^Differences and odds ratios between arms were calculated using the CMH method, stratified by pooled geographic region (Asia versus rest of world), prior definitive therapy (yes versus no), and investigator choice of chemotherapy (platinum with fluoropyrimidine versus platinum with paclitaxel) per IRT.
^c^Two-sided CMH test was stratified by pooled geographic region, prior definitive therapy, and investigator choice of chemotherapy per IRT.

^d^SD includes SD and non-CR/non-PD.

^e^Included patients with no postbaseline response assessment (Not Assessable) or assessment as Not Evaluable (NE) per RECIST v1.1.

## Supplementary Table S10. Efficacy outcomes in patients with PD-L1 TAP score ≥5% by TC score subgroups

|  | **TC score ≥1%** | | **TC score <1%** | |
| --- | --- | --- | --- | --- |
|  | **Tislelizumab plus chemotherapy**  **(*n* = 133)** | **Placebo plus chemotherapy**  **(*n* = 125)** | **Tislelizumab plus chemotherapy**  **(*n* = 39)** | **Placebo plus chemotherapy**  **(*n* = 59)** |
| Median OS, months (95% CI) | 17.2 (15.3-22.9) | 10.6 (8.6-14.2) | 25.1 (15.2-33.8) | 9.8 (8.0-12.3) |
| HR (95% CI)^a^ | 0.68 (0.51-0.90) | | 0.47 (0.28-0.79) | |
| Median PFS, months (95% CI) | 8.2 (7.0-10.0) | 5.5 (4.3-6.5) | 9.6 (5.5-15.0) | 5.0 (4.1-6.9) |
| HR (95% CI)^a^ | 0.52 (0.39-0.70) | | 0.45 (0.26-0.78) | |
| Objective response rate, *n* | 94 | 46 | 29 | 30 |
| % (95% CI)^b^ | 70.7 (62.2-78.2) | 36.8 (28.4-45.9) | 74.4 (57.9-87.0) | 50.8 (37.5-64.1) |
| Odds ratio, (95% CI)ᶜ | 4.05 (2.39-6.87) | | 3.05 (1.23-7.61) | |
| Two-sided CMH test *P* valueᵈ | < 0.0001 | | 0.0175 | |
| ORR difference, % (95% CI)ᶜ | 33.2 (21.6-44.7) | | 24.8 (6.1-43.4) | |

Data cutoff: 22 August 2024. Analysis includes patients with PD-L1 TAP score ≥5% stratified by TC score. ORR is unconfirmed and defined as proportion of patients with complete response or partial response per RECIST v1.1.

CI, confidence interval; CMH, Cochran-Mantel-Haenszel; HR, hazard ratio; ORR, objective response rate; OS, overall survival; PD-L1, programmed death-ligand 1; PFS, progression-free survival; TAP, Tumor Area Positivity; TC, tumor cell.

^a^HRs were calculated using Cox regression models with treatment as the covariate, stratified by investigator-chosen chemotherapy, region, and history of previous definitive therapy.

^b^Two-sided 95% CI was calculated using Clopper-Pearson method.

^c^Objective response rate differences and odds ratios between arms were calculated using the CMH method, stratified by pooled geographic region, prior definitive therapy, and investigator choice of chemotherapy.

^d^Two-sided CMH test was stratified by pooled geographic region, prior definitive therapy, and investigator choice of chemotherapy.

## Supplementary Table S11. Post-treatment subsequent anticancer therapies in patients with PD-L1 TAP score ≥5%

|  | **Tislelizumab plus chemotherapy (*n* = 172)** | **Placebo plus chemotherapy (*n* = 186)** |
| --- | --- | --- |
| Any subsequent anticancer therapy, *n* (%) | 106 (61.6) | 126 (67.7) |
| Radiotherapy | 37 (21.5) | 47 (25.3) |
| Procedure or surgery | 8 (4.7) | 8 (4.3) |
| Systemic therapy | 95 (55.2) | 114 (61.3) |
| Immunotherapy | 27 (15.7) | 44 (23.7) |

PD-L1, programmed death-ligand 1; TAP, Tumor Area Positivity.

## Supplementary Table S12. TRAEs leading to death (safety population) in patients with PD-L1 TAP score ≥5%.

| ***n* (%)** | **Tislelizumab plus Chemotherapy (*n* = 171)** | **Placebo plus Chemotherapy  (*n* = 185)** |
| --- | --- | --- |
| Patients with ≥1 TRAE leading to death | 5 (2.9) | 3 (1.6) |
| Gastrointestinal hemorrhage | 1 (0.6) | 0 (0.0) |
| Upper gastrointestinal hemorrhage | 1 (0.6) | 0 (0.0) |
| Pulmonary tuberculosis | 1 (0.6) | 0 (0.0) |
| Electrolyte imbalance | 1 (0.6) | 0 (0.0) |
| Respiratory failure | 1 (0.6) | 0 (0.0) |
| Pneumonia | 0 (0.0) | 1 (0.5) |
| Septic shock | 0 (0.0) | 1 (0.5) |
| Death^a^ | 0 (0.0) | 1 (0.5) |

Patients with multiple events for a given preferred term were counted only once for the preferred term.

Adverse events terms were coded using Medical Dictionary for Drug Regulatory Affairs (MedDRA) version 24.0.

TRAEs include TEAEs that were considered by the investigator to be related to study treatments or TEAEs with a missing causality. The death event due to disease progression of esophageal squamous cell carcinoma is requested to be reported as an AE if the death event occurred ≤30 days after the last dose of study drug per protocol. Those events are not included as TEAE leading to death.

^a^Reason not specified.

AE, adverse event; PD-L1, programmed death-ligand 1; TAP, Tumor Area Positivity; TEAE, treatment-emergent adverse event; TRAE, treatment-related adverse event.

## Supplementary Table S13. TRAEs leading to any treatment discontinuation **(safety population) in patients with PD-L1 TAP score ≥5%**

| ***n* (%)** | **Tislelizumab plus Chemotherapy (*n* = 171)** | **Placebo plus Chemotherapy  (*n* = 185)** |
| --- | --- | --- |
| Patients with ≥1 TRAE leading to treatment discontinuation | 55 (32.2) | 34 (18.4) |
| Peripheral sensory neuropathy | 16 (9.4) | 6 (3.2) |
| Hypoesthesia | 5 (2.9) | 4 (2.2) |
| Anemia | 4 (2.3) | 1 (0.5) |
| Neurotoxicity | 4 (2.3) | 3 (1.6) |
| Malaise | 3 (1.8) | 0 (0.0) |
| Pneumonitis | 3 (1.8) | 1 (0.5) |
| Stomatitis | 3 (1.8) | 0 (0.0) |
| Drug hypersensitivity | 2 (1.2) | 1 (0.5) |
| Gamma-glutamyltransferase increased | 2 (1.2) | 0 (0.0) |
| Acute kidney injury | 1 (0.6) | 0 (0.0) |
| Alanine aminotransferase increased | 1 (0.6) | 0 (0.0) |
| Alopecia | 1 (0.6) | 0 (0.0) |
| Alpha hydroxybutyrate dehydrogenase increased | 1 (0.6) | 0 (0.0) |
| Arthritis | 1 (0.6) | 0 (0.0) |
| Aspartate aminotransferase increased | 1 (0.6) | 0 (0.0) |
| Asthenia | 1 (0.6) | 0 (0.0) |
| Beta 2 microglobulin increased | 1 (0.6) | 0 (0.0) |
| Blood creatinine increased | 1 (0.6) | 1 (0.5) |
| Blood lactate dehydrogenase increased | 1 (0.6) | 0 (0.0) |
| Blood pressure increased | 1 (0.6) | 0 (0.0) |
| Deafness | 1 (0.6) | 0 (0.0) |
| Dyspnea | 1 (0.6) | 0 (0.0) |
| Electrolyte imbalance | 1 (0.6) | 0 (0.0) |
| Enteritis | 1 (0.6) | 0 (0.0) |
| Erythema | 1 (0.6) | 0 (0.0) |
| Hypotension | 1 (0.6) | 0 (0.0) |
| Immune-mediated dermatitis | 1 (0.6) | 0 (0.0) |
| Lip swelling | 1 (0.6) | 0 (0.0) |
| Mouth ulceration | 1 (0.6) | 0 (0.0) |
| Muscular weakness | 1 (0.6) | 0 (0.0) |
| Myalgia | 1 (0.6) | 0 (0.0) |
| Myositis | 1 (0.6) | 0 (0.0) |
| Neutrophil count decreased | 1 (0.6) | 1 (0.5) |
| Palmar-plantar erythrodysesthesia syndrome | 1 (0.6) | 3 (1.6) |
| Peripheral sensorimotor neuropathy | 1 (0.6) | 0 (0.0) |
| Platelet count decreased | 1 (0.6) | 1 (0.5) |
| Pulmonary tuberculosis | 1 (0.6) | 0 (0.0) |
| Rash | 1 (0.6) | 0 (0.0) |
| Renal impairment | 1 (0.6) | 2 (1.1) |
| Troponin I increased | 1 (0.6) | 0 (0.0) |
| Upper gastrointestinal hemorrhage | 1 (0.6) | 0 (0.0) |
| White blood cell count decreased | 1 (0.6) | 0 (0.0) |
| Acquired soft palate fissure | 0 (0.0) | 1 (0.5) |
| Agitation | 0 (0.0) | 1 (0.5) |
| Chills | 0 (0.0) | 1 (0.5) |
| Chronic kidney disease | 0 (0.0) | 2 (1.1) |
| Diarrhea | 0 (0.0) | 1 (0.5) |
| Fatigue | 0 (0.0) | 2 (1.1) |
| Hypoacusis | 0 (0.0) | 1 (0.5) |
| Lipase increased | 0 (0.0) | 1 (0.5) |
| Palpitations | 0 (0.0) | 1 (0.5) |
| Paresthesia | 0 (0.0) | 1 (0.5) |
| Pneumonia | 0 (0.0) | 1 (0.5) |
| Pneumonia aspiration | 0 (0.0) | 1 (0.5) |
| Sitophobia | 0 (0.0) | 1 (0.5) |
| Vomiting | 0 (0.0) | 1 (0.5) |

Patients with multiple events for a given preferred term were counted only once for the preferred term.

Adverse events terms were coded using Medical Dictionary for Drug Regulatory Affairs (MedDRA) version 24.0.

TRAEs include TEAEs that were considered by the investigator to be related to study treatments or TEAEs with a missing causality.

AE, adverse event; PD-L1, programmed death-ligand 1; TAP, Tumor Area Positivity; TEAE, treatment-emergent adverse event; TRAE, treatment-related adverse event.

## Supplementary Table S14. Overall summary of immune-mediated adverse events **(safety population) in patients with PD-L1 TAP score ≥5%**

| ***n* (%)** | **Tislelizumab plus Chemotherapy (*n* = 171)** | **Placebo plus Chemotherapy  (*n* = 185)** |
| --- | --- | --- |
| Patients with ≥1 immune-mediated AE | 73 (42.7) | 40 (21.6) |
| Immune-mediated AEs of ≥ grade 3 | 15 (8.8) | 4 (2.2) |
| Serious immune-mediated AEs | 12 (7.0) | 3 (1.6) |
| Immune-mediated AEs leading to dose modification of tislelizumab/placebo^a^ | 23 (13.5) | 3 (1.6) |
| Immune-mediated AEs leading to treatment discontinuation of tislelizumab/placebo | 8 (4.7) | 1 (0.5) |
| Immune-mediated AEs leading to death | 0 | 0 |
| Immune-mediated AEs treated with systemic corticosteroids | 24 (14.0) | 4 (2.2) |
| Immune-mediated AEs treated with immunosuppressants^b^ | 1 (0.6) | 1 (0.5) |
| Immune-mediated AEs treated with hormone therapies^c^ | 20 (11.7) | 5 (2.7) |
| Most frequently reported immune-mediated AEs by preferred term^d^ |  |  |
| Hypothyroidism | 23 (13.5) | 7 (3.8) |
| Rash | 21 (12.3) | 14 (7.6) |
| Pneumonitis | 12 (7.0) | 6 (3.2) |
| Hyperthyroidism | 7 (4.1) | 2 (1.1) |

For each row category, a patient with two or more adverse events in that category was counted only once.

^a^The types of dose modification included dose delay, infusion interruption, infusion rate decreased, and dose reduction for chemotherapy; and dose delay, infusion interruption, and infusion rate decreased for tislelizumab/placebo.

^b^Systemic glucocorticoids are not included in the immunosuppressants.

^c^Hormone therapies included thyroid replacement/anti-thyroid agents for hypothyroidism, hyperthyroidism, or thyroiditis, and insulin for type 1 diabetes mellitus.

^d^Most frequently reported immune-mediated AEs are those with incidence >3% in the tislelizumab plus chemotherapy arm.

AE, adverse event; PD-L1, programmed death-ligand 1; TAP, Tumor Area Positivity.

## Supplementary Table S15. Incidences of selected treatment-emergent adverse events over time **(safety population) in patients with PD-L1 TAP score ≥5%^a^**

| ***n/M* (%)** | **Tislelizumab plus chemotherapy (*n* = 171)** | **Placebo plus**  **chemotherapy  (*n* = 185)** |
| --- | --- | --- |
| Decreased appetite |  |  |
| ≤12 months | 70/171 (40.9) | 74/185 (40.0) |
| >12-24 months | 7/52 (13.5) | 0/22 (0) |
| >24-36 months | 1/29 (3.4) | 0/7 (0) |
| >36 months | 2/13 (15.4) | 0/1 (0) |
| Vomiting |  |  |
| ≤12 months | 37/171 (21.6) | 54/185 (29.2) |
| >12-24 months | 2/52 (3.8) | 1/22 (4.5) |
| >24-36 months | 1/29 (3.4) | 0/7 (0) |
| >36 months | 0/13 (0) | 0/1 (0) |
| Pruritus |  |  |
| ≤12 months | 20/171 (11.7) | 11/185 (5.9) |
| >12-24 months | 3/52 (5.8) | 1/22 (4.5) |
| >24-36 months | 2/29 (6.9) | 0/7 (0) |
| >36 months | 0/13 (0.0) | 0/1 (0) |
| Hypothyroidism |  |  |
| ≤12 months | 22/171 (12.9) | 7/185 (3.8) |
| >12-24 months | 1/52 (1.9) | 1/22 (4.5) |
| >24-36 months | 1/29 (3.4) | 1/7 (14.3) |
| >36 months | 0/13 (0) | 0/1 (0) |

^a^Any treatment-emergent adverse events with an incidence difference greater than 5% between the two arms at the interim analysis are included in the table.

*n* is the number of patients with at least one occurrence in each time interval.

*M* is the number of patients whose treatment-emergent period is within or beyond the time interval.

Percentages were calculated based on *M*.

PD-L1, programmed death-ligand 1; TAP, Tumor Area Positivity.

## Supplementary Figure S1. Changes at cycle 6 from baseline in EORTC QLQ-C30 and in EORTC QLQ-OES18 scores in patients with PD-L1 TAP score ≥5%

Data cutoff: 22 August 2024. Except for symptoms dysphagia, eating, and reflux domains, two-sided *P* value is for descriptive purpose only.

EORTC QLQ-C30, European Organisation for Research and Treatment of Cancer Quality of Life Questionnaire–Cancer 30 question module; EORTC QLQ-OES18, European Organisation for Research and Treatment of Cancer Quality of Life Questionnaire–Oesophageal Cancer 18 question module; LS least squares; PD-L1, programmed death-ligand 1; TAP, Tumor Area Positivity.

## Supplementary Figure S2. Changes at cycle 8 from baseline in EORTC QLQ-C30 and in EORTC QLQ-OES18 scores in patients with PD-L1 TAP score ≥5%

Data cutoff: 22 August 2024. Two-sided *P* value is for descriptive purpose only.

EORTC QLQ-C30, European Organisation for Research and Treatment of Cancer Quality of Life Questionnaire–Cancer 30 question module; EORTC QLQ-OES18, European Organisation for Research and Treatment of Cancer Quality of Life Questionnaire–Oesophageal Cancer 18 question module; LS least squares; PD-L1, programmed death-ligand 1; TAP, Tumor Area Positivity.
